# Potentiation of Phase Variation in Multiple Outer-Membrane Proteins During Spread of the Hyperinvasive *Neisseria meningitidis* Serogroup W ST-11 Lineage

**DOI:** 10.1093/infdis/jiz275

**Published:** 2019-05-23

**Authors:** Luke R Green, Neelam Dave, Adeolu B Adewoye, Jay Lucidarme, Stephen A Clark, Neil J Oldfield, David P J Turner, Ray Borrow, Christopher D Bayliss

**Affiliations:** 1 Department of Genetics and Genome Biology, University of Leicester, Leicester; 2 Meningococcal Reference Unit, Public Health England, Manchester Royal Infirmary, Manchester; 3 School of Life Sciences, University of Nottingham, Nottingham, United Kingdom

**Keywords:** *Neisseria meningitidis*, serogroup W, phase variation, simple sequence repeat, NadA, Opa

## Abstract

**Background:**

Since 2009, increases in the incidence of invasive meningococcal disease have occurred in the United Kingdom due to a sublineage of the *Neisseria meningitidis* serogroup W ST-11 clonal complex (hereafter, the “original UK strain”). In 2013, a descendent substrain (hereafter, the “2013 strain”) became the dominant disease-causing variant. Multiple outer-membrane proteins of meningococci are subject to phase-variable switches in expression due to hypermutable simple-sequence repeats. We investigated whether alterations in phase-variable genes may have influenced the relative prevalence of the original UK and 2013 substrains, using multiple disease and carriage isolates.

**Methods:**

Repeat numbers were determined by either bioinformatics analysis of whole-genome sequencing data or polymerase chain reaction amplification and sizing of fragments from genomic DNA extracts. Immunoblotting and sequence-translation analysis was performed to identify expression states.

**Results:**

Significant increases in repeat numbers were detected between the original UK and 2013 strains in genes encoding PorA, NadA, and 2 Opa variants. Invasive and carriage isolates exhibited similar repeat numbers, but the absence of *pilC* gene expression was frequently associated with disease.

**Conclusions:**

Elevated repeat numbers in outer-membrane protein genes of the 2013 strain are indicative of higher phase-variation rates, suggesting that rapid expansion of this strain was due to a heightened ability to evade host immune responses during transmission and asymptomatic carriage.

## Teaser

Increased numbers of repeats were observed in multiple phase-variable genes encoding outer-membrane proteins during evolution of the *Neisseria meningitidis* hypervirulent MenW ST-11 clonal complex, indicative of a heightened potential for immune evasion during transmission and carriage of this pathogen.

Over the last 2 decades, multiple countries have experienced a significant increase in the incidence of endemic invasive meningococcal disease (IMD) due to *Neisseria meningitidis* serogroup W (MenW) isolates [1]. This altered epidemiology was driven by the emergence of ST-11 clonal complex (cc11) lineage 11.1 within South America, followed by subsequent spread to the United Kingdom and Europe [2]. IMD due to this lineage has a high case-fatality rate and often atypical clinical presentation [3]. Genomic surveillance within the United Kingdom has revealed a diverse population structure, with initial emergence of the MenW cc11 strain (hereafter, the “original UK strain”) in 2009, followed by the appearance of a novel descendant in 2013 (hereafter, the “2013 strain”) that rapidly became the major cause of MenW-related IMD in the United Kingdom, Europe, and Canada [4]. In a 2015–2016 cross-sectional study of university students, Oldfield et al observed rapid increases in carriage of the MenW cc11 2013 strain but not the original UK strain, suggestive of a higher propensity for transmission and persistent carriage of the 2013 strain [5].

Asymptomatic carriage of *N. meningitidis* within the nasopharynx is required before initiation of IMD. Carriage rates rise from approximately 4.5% in infants to approximately 24% in individuals aged 19 years [6], with rates of up to 60% in university student residences [7]. IMD can manifest as sepsis and/or meningitis and occurs in individuals without protective bactericidal antibodies, when meningococci invade the bloodstream and/or cross the blood-brain barrier. Changes occur frequently in meningococcal genomes during infections. Aside from mutations that alter capsule expression, no genetic changes have been directly associated with the invasive phenotype across multiple IMD cases [8]. Lees et al observed limited genetic adaptation between paired meningococcal isolates from the blood versus the cerebral spinal fluid, suggesting that the invasive phenotype is established during transition from asymptomatic carriage on mucosal surfaces to systemic sites of infection [9].

Meningococcal phenotypic variation occurs frequently as a result of phase variation (PV) mediated by slipped-strand mispairing of simple sequence repeats (SSRs) [10]. In meningococci, the SSRs typically consists of polyG or polyC tracts or tandemly arranged tetranucleotide or pentanucleotide repeats present in the reading frame or promoters of genes such that changes in repeat number cause alterations in translation or transcription. These SSRs mutate at rates of >1 × 10^-5^ mutations/division, with repeat number and mismatch repair being key determinants of switching rate [11–13]. Approximately 50 meningococcal genes can undergo PV, with the majority encoding outer-membrane proteins (OMPs) with functions such as adhesion and iron acquisition [14]. While changes in SSRs during persistent asymptomatic carriage can lead to a reduction in expression of OMPs [15], there has been no extensive comparison of SSRs or PV states between carriage and invasive isolates of a single clonal complex.

This study compares the repeat numbers and expression states of phase-variable OMPs for the MenW ST-11 lineage between (1) the original UK and 2013 strains and (2) carriage and invasive isolates. We demonstrate a heightened potential for PV of multiple OMPs in the 2013 strain and reduced expression of PilC proteins in invasive isolates. These data highlight the potential importance of meningococcal SSR variation for switching from asymptomatic carriage to IMD and how evolution of SSRs may contribute to the spread of meningococcal strains.

## METHODS

### Acquisition of Genome Sequences

MenW cc11 whole-genome sequences (WGS) were extracted from the PubMLST *Neisseria* database (available at: https://pubmlst.org/bigsdb?db=pubmlst_neisseria_isolates). Invasive isolates recovered from July 2010 to October 2017 were collected by interrogating the Meningitis Research Foundation Meningococcal Genome Library for clonal complex ST-11 and capsule group W. Carriage isolates were selected from either a 2010–2012 Novartis multicenter carriage study [16] or a 2015–2016 University of Nottingham carriage study [17].

### Analysis of the Repeat Tracts of OMPs, *pilC* and *opa* Genes

Sequence contigs were annotated using PROKKA (version 1.11), with M25419 (GCA_001697985.1) as a reference, followed by repeat number determination with Phasome*It* [18]. Incomplete repeat tracts were rebuilt by mapping raw FASTQ reads to the M25419 WGS, using BWA (version 0.7.16a), followed by extraction of SSR-specific reads with an adjacent, conserved 1-kb trap sequence. Extracted reads were assembled using SPAdes (version 3.9.0), and repeat numbers were manually determined [19]. For a subset of isolates, repeat numbers were examined by GeneScan analysis of polymerase chain reaction (PCR) products generated using fluorescently labeled primers spanning the SSR (Supplementary Table 1 and [15]). Fragment sizes were determined and converted into repeat numbers, using Peakscanner (version 1.0), BioEdit (version 7.2.5), and Microsoft Excel.

### Measurement of Protein Expression Levels

For immunoblots, heat-killed bacterial suspensions were spotted onto a nitrocellulose membrane. For Western blots, bacterial lysates produced by heating cell suspensions in 1× sodium dodecyl sulfate loading buffer at 65°C for 15 minutes were subjected to polyacrylamide gel electrophoresis and transferred to polyvinylidene difluoride membranes. Membranes were incubated overnight in 10 mM Tris (pH 7.5), 150 mM NaCl, 0.05% Tween 20, phosphate-buffered saline, and 5% milk. Membranes were then probed with a specific primary antibody (ie, an anti-Opa mouse polyclonal antisera [20], PorA-specific mouse monoclonal antibody P1.5 [21], or a PilC-specific mouse monoclonal antibody kindly provided by Christopher Thomas), followed by a goat anti-mouse IgG conjugated to either horseradish peroxidase or alkaline phosphatase. Findings were visualized using either an enhanced chemiluminescence kit (Biological Industries) or a BCIP/NBT substrate solution.

### Statistical Analysis

For each gene and group of isolates, repeat numbers were separated into those that were less than or equal to the modal repeat and those that were greater than the modal repeat (derived from an analysis of all isolates for each gene). Expression states (as defined elsewhere [15]) were similarly split into 2 nonoverlapping categories (ie, ON or OFF; and HIGH or INTERMEDIATE/LOW). These numbers were sorted into a contingency table and analyzed using a 2-tailed Fisher exact test in Microsoft Excel. A *P* value of ≤ .05 was considered statistically significant.

## RESULTS

### Allelic Variation in Phase-Variable Single-Copy OMPs (scOMPs) of ST-11 MenW Carriage and Invasive Isolates

We assessed the association of PV with IMD by focusing on the United Kingdom cc11 MenW strains, using 636 United Kingdom invasive disease isolates (obtained between 2010 and 2017) and 101 United Kingdom carriage isolates (ie, 47 and 54 isolates obtained from university students in a 2010–2011 longitudinal study and a 2015–2016 cross-sectional study, respectively). Isolates of the MenW cc11 original UK strain and 2013 strain were identified through a phylogenetic analysis of the core genome (ie, the N. meningitidis cgMLST v1.0 scheme). The invasive isolates were evenly distributed between the original UK strain (315 [49.5%]) and the 2013 strain (321 [50.5%]; Supplementary Data File 1). A similar distribution was observed for the carriage isolates.

Gene sequences for 7 scOMP PV genes were extracted from WGS data. A single allele was dominant among the 737 invasive and carriage isolates for 6 loci (*fetA*, allele 13: 656 isolates [89%]; *nadA*, allele 5: 729 [99%]; *porA*, allele 1: 685 [93%]; *hmbR*, allele 1: 693 [94%]; *mspA*, allele 1: 733 [99%]; and *nalP*, allele 2: 494 [67%]; Supplementary Table 2). Because *hpuA* was frequently broken owing to poor assembly across the repeat tract, a BLAST search was performed with a truncated sequence lacking the repeat tract and approximately 57 N-terminal nucleotides. Of the 688 isolates positive for the truncated *hpuA*, 294 (43%) contained allele 167, which is associated with the original UK strain, and 326 (47%) contained allele 175, which is associated with the 2013 strain [4]. Allele numbers were not extracted for the *pilC* and *opa* loci, owing to poor assembly of long-repeat tracts and highly similar multicopy loci. No indels were detected outside of the repeat tracts for PV genes with intragenic tracts; therefore, the repeat number correlates with the expression state for all alleles (Supplementary Figure 1).

### Repeat Numbers Vary Significantly for Multiple Phase-Variable Genes Between the Original UK Strain and the 2013 Strain

**Figure 1.**
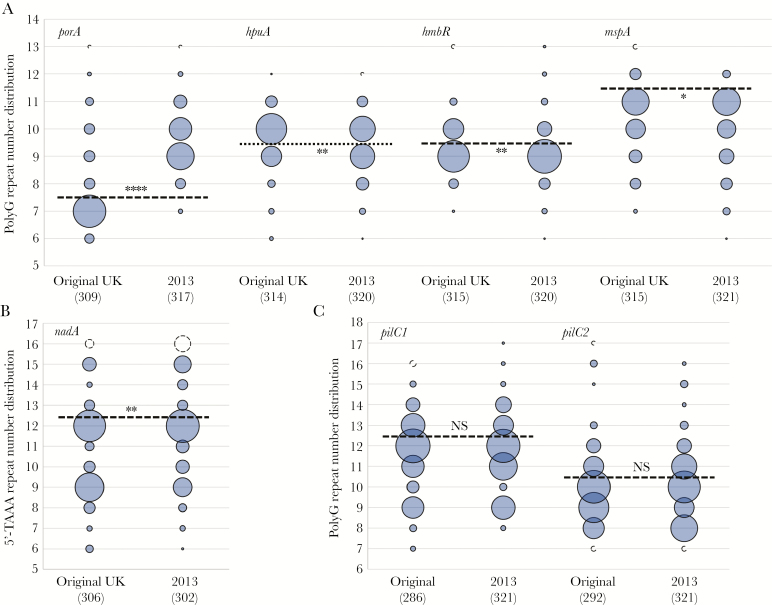
Significant changes in repeat number for 5 phase-variable single-copy outer-membrane protein (scOMP) genes but not *pilC* genes between the *Neisseria meningitidis* serogroup W (MenW) clonal complex 11 original UK and 2013 strains. Repeat numbers were determined for these phase-variable genes by bioinformatics analyses of whole-genome sequencing data for multiple disease isolates of the original UK and 2013 strains of the MenW ST-11 South American strain lineage. The total number of isolates of each strain type is shown in parentheses. Shaded bubbles represent the proportion of isolates with a particular repeat number. Non-shaded bubbles indicate the proportion of isolates with ≥ or ≤ for high and low repeat numbers, respectively. Dashed line, modal repeat number (note that the line is placed above the modal number); dotted line (*hpuA*), separates into different groups isolates with 9 and 10 repeats, which are the major repeat numbers associated with OFF and ON expression states, respectively; asterisks above or below the dotted line indicate significant differences in the proportion of isolates with repeat numbers above or below the mode, respectively, for the 2013 strain. NS, not significant. *P ≤ .05, **P ≤ .01, and ****P ≤ .0001.

Repeat numbers in 7 phase-variable scOMPs and the 2 *pilC* loci were determined for >93% of 737 MenW ST-11 isolates (Supplementary Data File 1). Repeat numbers in 6 scOMP genes and the *pilC* loci were corroborated by GeneScan analyses for 77%–100% of a select group of isolates, suggesting that WGS analysis has a relatively high degree of accuracy (Supplementary Table 3). Modal repeat numbers were calculated for all isolates for each gene and used for detecting shifts in hypermutable tract length during evolution of the MenW ST-11 strain. No significant differences were observed in repeat numbers for *fetA*, *nalP*, *pilC1*, and *pilC2* between invasive isolates of the MenW cc11 original UK strain and the 2013 strain (Supplementary Figure 2). For the polyG tract in *porA*, significantly more isolates had repeat numbers of >7 in the 2013 strain (314 of 317 [99%]) as compared to the original UK strain (93 of 309 [30%]; *P* < .0001; Figure 1A). Similarly, a significantly larger number of isolates had *nadA* 5′-TAAA repeat numbers greater than the mode (12) in the 2013 strain (89 of 302 [30%]) as compared to the original UK strain (50 of 306 [16%]; *P* = .0061; Figure 1B). For both genes, differences were observed in a wide range of repeat numbers greater than the mode (eg, 9, 10, and 11 repeats for *porA*). Contrastingly, repeat numbers for the *hpuA* polyG, *hmbR* polyG, and *mspA* polyC repeat tracts were significantly less than the mode in the 2013 strain as compared to the original UK strain (163 of 320 [51%] vs 105 of 314 [33%] for *hpuA* [*P* = .0016], 259 of 320 [81%] vs 213 of 315 [68%] for *hmbR* [*P* = .0072], and 308 of 321 [96%] vs 282 of 315 [90%] for *mspA* [*P* = .025]; Figure 1A, Figure 1C). For these genes, the reduction was by a single repeat, commensurate with a change in expression state (eg, *hpuA* exhibits a decrease in isolates with 10 repeats and an increase in those with 9 repeats, which corresponds to an ON-to-OFF expression state switch).

**Figure 2.**
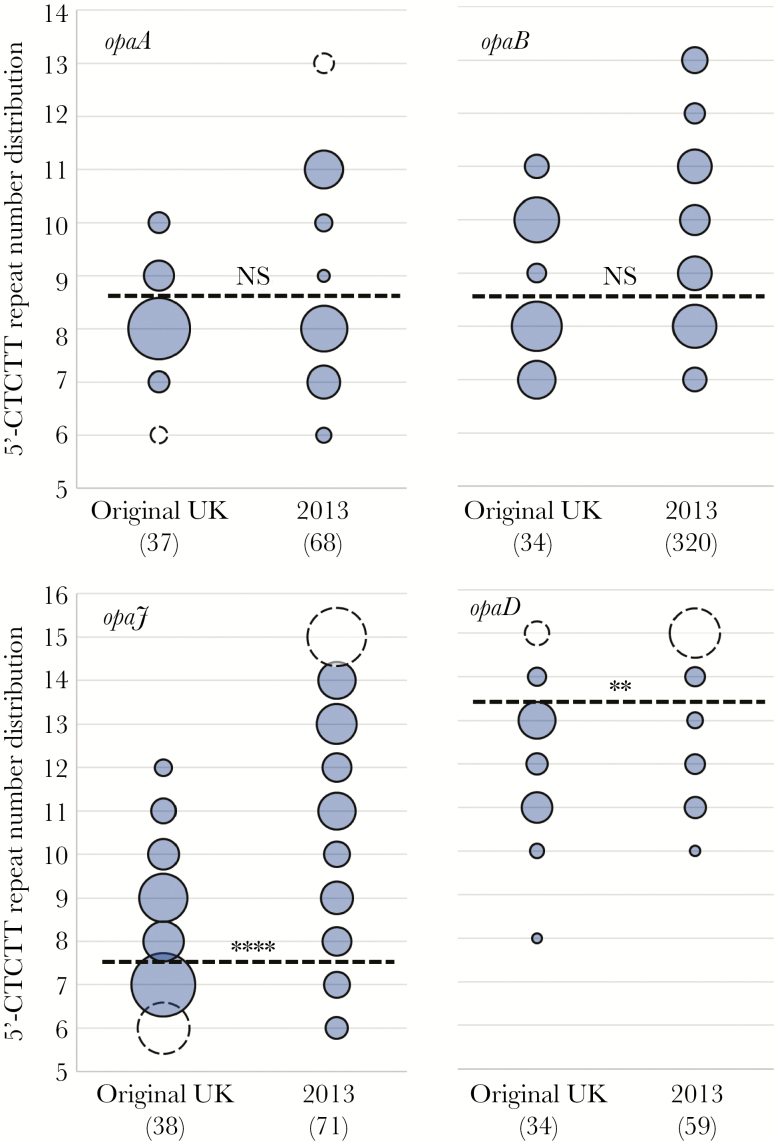
Increases in repeat number for the 4 phase-variable *opa* genes between the *Neisseria meningitidis* serogroup W (MenW) clonal complex 11 original UK and 2013 strains. Repeat numbers were determined for the 4 *opa* loci by polymerase chain reaction fragment analysis of genomic DNA extracts for multiple disease isolates of the original UK and 2013 strains of the MenW ST-11 South American strain lineage (total numbers of isolates of each type are shown in parentheses). The bubbles and dashed lines are as described for Figure 1. NS, not significant. **P ≤ .01 and ****P ≤ .0001.

The 5′-CTCTT repeat tracts of the 4 *opa* genes were analyzed by PCR fragment analysis in a subset of MenW ST-11 strains (54 carriage and 121 invasive isolates; Supplementary Data File 1). A significantly higher proportion of 2013 strain isolates than original UK strain isolates harbored repeat numbers exceeding the mode for the *opaJ* (64 of 71 [90%] vs 18 of 38 [47%]; *P* ≤ .0001) and *opaD* (40 of 59 [68%] vs 8 of 34 [24%]; *P* = .003; Figure 2) loci. For *opaJ*, there was a striking increase in the number of isolates with ≥13 repeats (Figure 2). No significant differences were observed in repeat numbers greater the mode for *opaA* or *opaB*, but both genes exhibited higher numbers of isolates with ≥11 repeats for the 2013 strain (Figure 2).

### scOMPs and *pilC* Loci Differ in Repeat Numbers Between Invasive and Carriage Isolates

Repeat numbers were also compared between invasive and carriage MenW ST-11 isolates for both strains (Supplementary Table 4). No significant differences were detected for *hpuA* or *hmbR* in either substrain. Significant differences were detected for *nalP*, *mspA, pilC1*, and *pilC2* with the original UK strain, but these data sets may be influenced by multiple longitudinal isolates from the same carrier. For the 2013 strain, a significantly higher proportion of carriage isolates, as compared to invasive isolates, had repeat numbers that were greater than the mode for *nadA*, *fetA*, and *pilC1* (eg, 57% of carriage isolates and 23% of invasive isolates had *pilC1* tracts of >12 repeats; *P* = .0001; Supplementary Table 4). Similar results were obtained for a comparison of invasive isolates from either the East Midlands only (47) or a random selection (54) against the 54 carriage isolates for the 2013 strain (data not shown).

A similar comparison of invasive and carriage MenW isolates for both substrains was also made with *opa* loci repeat numbers (Supplementary Table 4). Only *opaD* showed a significant difference in the original UK strain; however, this analysis involved only 5 carriage isolates. For the 2013 strain, the *opaA* repeat numbers were significantly higher for carriage isolates than for invasive isolates (80% vs 37% had *opaA* tracts of >8 repeats; *P* = .011). No significant differences were observed for the other *opa* loci.

### Differential Expression of Phase-Variable Genes Between United Kingdom MenW ST-11 Strains

**Figure 3.**
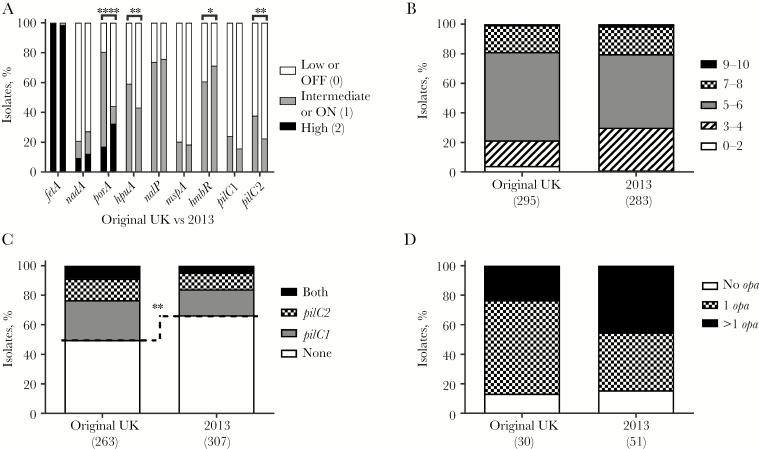
Isolates of the *Neisseria meningitidis* serogroup W (MenW) 2013 strain exhibit a high prevalence of phase-variable ON states associated with multiple single-copy outer-membrane proteins (scOMPs) but not PilC proteins. Expression states for each phase-variable gene were predicted from repeat numbers (see text); transcriptional simple sequence repeats (SSRs; *porA*, *fetA*, and *nadA*) were coded as low (0), intermediate (1), and high (2) expression; translational SSRs were coded as ON (1) or OFF (0). Phasotypes were derived from these expression states for the 7 scOMPs, 2 PilC proteins, and 4 Opa proteins. For the scOMPs, phasotypic scores were derived by simple addition of the expression states of each phasotype. *A*, Proportion of isolates in each expression state for the MenW clonal complex 11 original UK strain (left bar) versus the 2013 strain (right bar). *B*, scOMP combinatorial phasotypic expression scores (10 [2 + 2 + 2 + 1 + 1 + 1 + 1], is the maximum score for the 7-gene scOMP phasotype). *C*, PilC phasotypes (PilC1-PilC2). D, Combinatorial Opa phasotypic expression scores (a score of 1 indicates expression of only one of the 4 *opa* loci). Significance values were obtained using the number of isolates with either a particular expression state or phasotypes/phasotypic scores above and below the dotted line. *P ≤ .05, **P ≤ .01, and ****P ≤ .0001.

PV gene expression states and phasotypes (ie, combinations of expression states of multiple genes) were predicted from gene sequences to determine whether specific PV states were associated with each MenW ST-11 substrain. Repeat-mediated changes in PorA protein levels were confirmed by immunoblotting (Supplementary Figure 3) and were associated with a significant shift to lower PorA expression in the 2013 strain as compared to the original UK strain (171 of 306 [53%] vs 59 of 305 [19%]; *P* < .0001; Figure 3A). Opposing shifts in expression of the iron-acquisition proteins were detected, with the 2013 isolates exhibiting higher expression of HmbR (228 of 320 [71%] vs 191 of 315 [62%]; *P* = .0056) but lower expression of HpuA (138 of 320 [43%] vs 186 of 314 [59%]; *P* < .0001; Figure 3A). A significant decrease in PilC2 expression was also detected in the 2013 strain, accompanied by a minor, nonsignificant shift to lower PilC1 expression. No significant changes were detected in expression of other scOMPs. Examination of multigene phasotypes detected no significant changes in the phasotypic scores of the 7 scOMP phasotypes between the 2013 strain and the original UK strain (Figure 3B) but revealed a significant shift to a loss of expression of both PilC proteins (ie, a 00 phasotype; Figure 3C). The loss of expression of both PilC proteins for the 00 phasotype was confirmed by Western blotting in a subset of isolates (Supplementary Figure 3).

**Figure 4.**
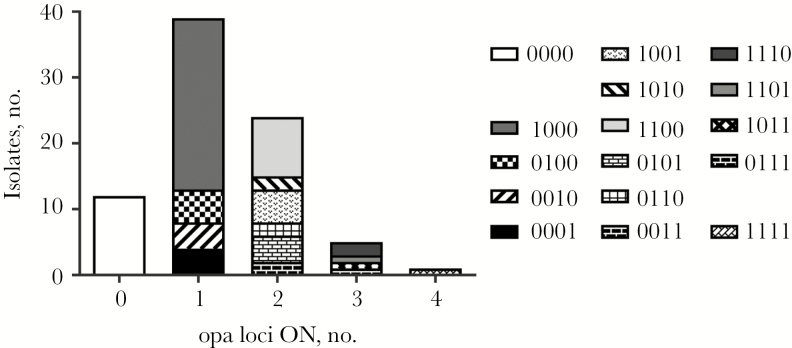
Predominance of expression of a single Opa protein and of OpaA in *Neisseria meningitidis* serogroup W (MenW) ST-11 isolates. Expression states for the 4 *opa* loci were predicted from repeat numbers for 121 MenW ST-11 isolates and converted into a 4-gene phasotype (OpaA-OpaB-OpaD-OpaJ), with 1 and 0 indicating ON and OFF states, respectively (eg, 1-0-0-0 indicates expression of OpaA but no other Opa proteins). The Opa designations refer to the following NMB encoding *opa* loci in the MC58 genome sequence: A, NMB0442; B, NMB1636; D, NMB1465; and J, NMB0926.

Expression states and phasotypes were predicted from repeat numbers for the 4 *opa* loci and confirmed in a subset of strains by Western blotting (Supplementary Figure 3). Whereas all 16 potential phasotypes were detected, the majority of invasive isolates (39 of 81 [48%]) expressed a single Opa protein, with a predominance of the OpaA-only phasotype (ie, 1000; 26 of 81 isolates [32%]; Figure 4). In contrast, expression of 0 or all 4 Opa proteins was infrequent (12 of 81 isolates [15%] and 1 of 81 [1%], respectively). The 2013 strain exhibited a nonsignificant trend toward higher expression of multiple Opa proteins than the original UK strain (23 of 51 isolates [45%] vs 7 of 30 [23%]; *P* = .1219; Figure 3D).

### Lower-Level PilC Expression Is Associated With IMD

**Figure 5.**
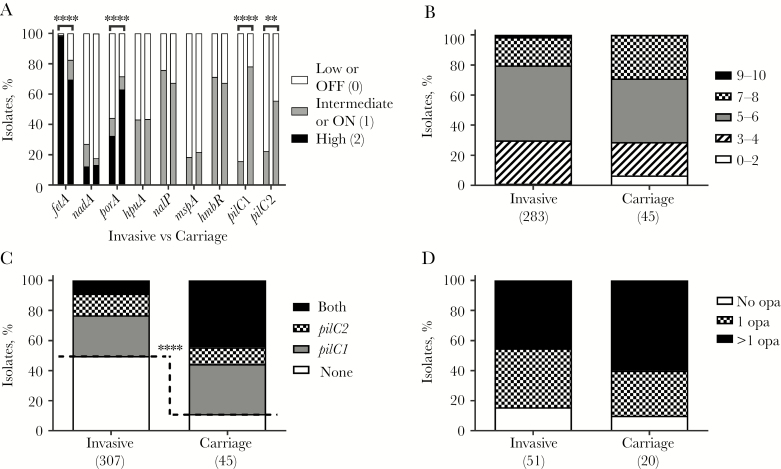
Lower expression of PilC proteins but not other phase-variable proteins in invasive isolates of the *Neisseria meningitidis* serogroup W (MenW) clonal complex 11 2013 strain. This figure uses the same disease-associated strains as in Figure 2 but shows a comparison to an analysis of the phase-variable genes of 46 carriage isolates of the 2013 strain from the 2015–2016 cross-sectional carriage study performed at the University of Nottingham. *A*, Individual gene expression states. *B*, Single-copy outer-membrane protein combinatorial phasotypic expression scores. *C*, PilC phasotypes (PilC1-PilC2). *D*, Combinatorial Opa phasotypic expression scores. **P ≤ .01 and ****P ≤ .0001.

Differential expression between invasive and carriage isolates was investigated using the 2013 strain. Significant differences in expression were only detected for *fetA, porA*, and *pilC*. The FetA expression state was HIGH in a significantly greater proportion of invasive isolates than carriage isolates (314 of 320 [98%] vs 32 of 46 [70%]; *P* < .0001; Figure 5A). Conversely, the PorA expression state was HIGH in a significantly greater proportion of carriage isolates than invasive isolates (37 of 46 [80%] vs 99 of 306 [32%]; *P* < .0001; Figure 5A). The expression state of both PilC1 and PilC2 was ON in a significantly greater proportion of carriage isolates (36 of 46 [78%] and 25 of 45 [56%], respectively) than in invasive isolates (49 of 311 [16%; *P* < .0001] and 71 of 316 [22%; *P* < .0001], respectively; Figure 5A). No significant differences were detected in scOMP phasotypes (Figure 5B) or for expression of multiple Opa proteins (Figure 5D). Contrastingly, the phasotype associated with no expression of PilC (ie, phasotype 00) was present at a significantly higher frequency in invasive isolates than carriage isolates (204 of 307 [66%] vs 5 of 45 [11%]; *P* < .0001; Figure 5C). These 2 findings were also significant if the carriage isolates were compared to invasive isolates from the East Midlands or to a random selection (Supplementary Figure 4).

### scOMP Expression Decreases Over Time During Long-term Carriage

**Figure 6.**
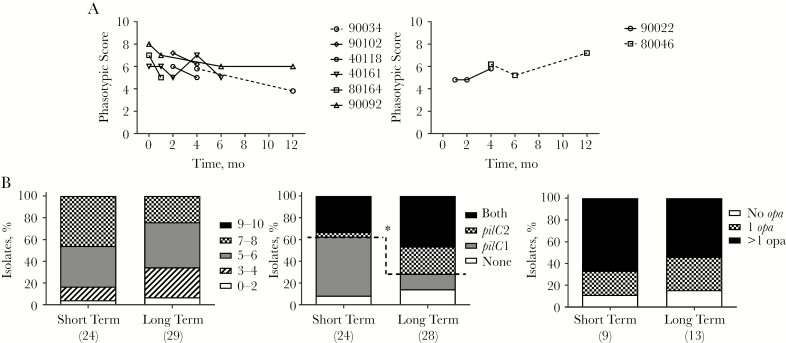
Reductions in the combinations of expressed single-copy outer-membrane proteins (scOMPs) and a switch to PilC2 expression during long-term *Neisseria meningitidis* serogroup W (MenW) ST-11 carriage. *A*, Changes in the scOMP phasotypic score over 12 months of persistent carriage as determined from analysis of a single isolate per time point for 8 longitudinal carriers derived from a 2010–2011 carriage study involving university students. *B*, Comparison of short- and long-term carriage isolates (see text) from the 2015–2016 carriage study in university students. See Figure 2 for derivation of scOMP and Opa phasotypic scores and PilC phasotypes. *P ≤ .05.

The 2 carriage cohorts were examined for evidence of changes in PV expression states during persistent carriage. The 2010–2011 study included 47 longitudinal nasopharyngeal isolates of the MenW original UK strain from 18 carriers with asymptomatic carriage for up to 12 months. Analysis of scOMP phasotypic scores identified 6 carriers with decreases in these combinatorial OMP expression states, 2 carriers with an increase, and 10 carriers with no consistent temporal changes (Figure 6A). Increases or decreases involved changes in expression of only 1 or 2 phase-variable OMPs.

The cross-sectional 2015–2016 carriage study consisted of 3 collection times. Assuming that carriage was acquired soon after arrival on campus in late September or was already present, we split the 54 carriers into short-term (December isolates) and long-term (early September/March isolates) carriage groups. There was a significant increase in PilC2 expression within long-term carriers as compared to short-term carriers (PilC phasotypes 01 and 11, 20 of 28 [71%] vs 9 of 24 [37%]; Figure 6B) and a trend toward lower expression of scOMPs in long-term carriers as compared to short-term carriers (phasotypic score of ≤6, 22 of 29 [76%] vs 13 of 24 [54%]; Figure 6B).

## Discussion

PV enables rapid adaptation to strong selective pressures and can mediate escape of bactericidal antibodies targeting specific surface antigens in vitro [22]. Adaptation by PV is influenced by the switching rate, with higher rates conferring a competitive advantage in both in vitro assays and in silico models [13, 22, 23]. We have explored evolution of the phase-variable genes in the MenW cc11 strain in the United Kingdom between 2009 and 2017 and the contributions of PV to the transition from asymptomatic carriage to invasive disease.

We have observed that there is a significant bias for higher repeat numbers for 4 OMPs (ie, PorA, NadA, OpaD, and OpaJ) in the MenW cc11 2013 strain as compared to the original UK strain. The PorA, NadA, and Opa proteins are highly immunogenic, and therefore the ability to evade antibody responses against these antigens may be critical during mucosal colonization and expansion. Repeat number is a key determinant of the PV rate; thus, our results suggest that higher PV rates for these surface proteins have evolved in the new strain. Since 2013, the number of IMD cases in the United Kingdom caused by the 2013 strain has increased more rapidly than the number associated with the original UK strain [4]. Additionally, a significant expansion of carriage of the 2013 strain but not the original UK strain was observed over a few months in a student population [17]. These findings indicate that the 2013 strain has a higher transmission rate than the original UK strain. The differences in phenotype of these strains have been attributed to allelic variation in HpuAB (one of 2 phase-variable hemoglobin binding proteins present in these strains) and 3 type IV pilus biogenesis genes and/or to a frameshift in *mtrA*, a transcriptional regulator of NadA expression [4]. We speculate that the heightened transmissibility may be due in part to higher PV rates enabling escape of antigen-specific or weakly cross-reactive antibodies during initial colonization of partially immune individuals or during persistence in and, hence, ongoing transmission from asymptomatic carriers. The former may be dominant, because longitudinal carriage was only weakly associated with PV-mediated expression changes. A previous study by Richardson et al [13] detected higher PV rates due to mismatch repair mutations in epidemic serogroup A isolates from the meningitis belt and similarly concluded that elevated PV rates enhance transmissibility in populations with high levels of strain-specific carriage.

Significantly higher repeat numbers were detected in carriage as compared to invasive isolates of the 2013 strain for *fetA*, *nadA*, *pilC1*, and *opaA*. While these results may be partially confounded by clonal expansion in the University of Nottingham carriage cohort, these results suggest that heightened PV rates may contribute to long-term persistence in the upper respiratory tract, possibly by facilitating immune evasion. However, the significant difference for *pilC1* is mainly due to the shift from 13 to 12 repeats that is associated with a switch from an ON to an OFF expression state in invasive isolates and, therefore, may reflect a requirement for a loss of function of this gene (see below) during IMD, rather than higher PV rates. The absence of differences in this analysis for the *porA*, *opaD*, and *opaJ* repeat numbers may suggest that the heightened PV in the 2013 strain, compared with the original UK strain, increases the propensity for both IMD and carriage and not for carriage alone. Because IMD is thought to occur soon after strain acquisition, the heightened PV rates of these genes may have a much stronger effect on initial colonization of carriers than on persistence, such that there is no difference between the carriage and invasive states.

Another key observation was for lower PV-mediated expression of PilC and multiple Opa proteins in disease isolates (93% of invasive isolates with source information were from blood samples). The type IV pilus is required for colonization of the microvasculature during IMD [24]. Because PilC mutants express low levels of pili on the bacterial surface, there may be a strong selection for PV-mediated switching OFF of PilC expression as meningococci detach from initial reservoirs of infection within the peripheral microvasculature and undergo rapid replication in the blood.

Our study focused on analysis of multiple isolates of a single hypervirulent clone from nonoverlapping sets of carriers and patients. We assume that our findings are representative of the switches in PV state occurring during transition from asymptomatic carriage to IMD in individuals. Previously, repeat numbers and expression states of 2 PV genes were found to be identical for DNA extracted from a clinical sample and the isolated disease-causing strain of individual patients, an indication that PV during strain isolation is infrequent [25]. Nevertheless, there is a potential that our study is not representative of actual disease processes and could be partially confounded by PV during strain isolation (as observed elsewhere [8]). Further studies of PV in both models of infection and with more complex epidemiological samples are required to address these issues.

In summary, our results highlight the genomic flexibility of meningococci and the potential for genetic variation to alter the course of an epidemic. We speculate that a small number of genetic alterations in the MenW cc11 2013 strain, including the observed alterations in repeat numbers of PV genes, has prolonged the persistence of this hypervirulent lineage in the United Kingdom, with a consequent increase in the number of disease cases.

## Supplementary Data

Supplementary materials are available at *The Journal of Infectious Diseases* online. Consisting of data provided by the authors to benefit the reader, the posted materials are not copyedited and are the sole responsibility of the authors, so questions or comments should be addressed to the corresponding author.

jiz275_suppl_Supplementary_Data_File

jiz275_suppl_Supplementary_Data_Figure_1

jiz275_suppl_Supplementary_Data_Figure_2

jiz275_suppl_Supplementary_Data_Figure_3

jiz275_suppl_Supplementary_Data_Figure_4

jiz275_suppl_Supplementary_Data_Tables

## Notes

***Acknowledgments.*** The PilC monoclonal antibody was a kind gift of Christopher E. Thomas from the University of North Carolina at Chapel Hill. This publication made use of the Meningitis Research Foundation Genome Library, developed by Public Health England, the Wellcome Trust Sanger Institute, and the University of Oxford, a project funded by Meningitis Research Foundation. This study also made use of the Neisseria Multi Locus Sequence Typing website (available at: https://pubmlst.org/neisseria/), hosted by the University of Oxford and developed with funding from the Wellcome Trust and European Union [26].

Experiments and bioinformatics and statistical analyses were performed by L. R. G., A. A., and N. D. N. J. O., J. L., and S. A. C. provided meningococcal isolates. Experimental design was conceived by N. J. O., D. P. J. T., R. B., and C. D. B. The manuscript was written by L. R. G. and C. D. B. All authors commented on the final manuscript.

***Disclaimer.*** The funders had no role in study design, data collection, and interpretation or the decision to submit the work for publication.

***Financial support.*** This work was supported by the Medical Research Council (MR/M020193/1).

***Potential conflicts of interest.*** C. D. B. reports grants from GlaxoSmithKline, Sanofi Pasteur MSD, and Roche, outside the submitted work. R. B. and J. L. report grants for contract research on behalf of Public Health England (formerly the Health Protection Agency) for GlaxoSmithKline, Novartis Vaccines, Pfizer, and Sanofi Pasteur MSD, outside the submitted work. J. L. and S. A. C. have performed contract research on behalf of Public Health England for GlaxoSmithKline and Pfizer. All other authors report no potential conflicts.

Presented in part: International Pathogenic Neisseria Conference, Asilomar, California, September 2018.

## References

[1] Mustapha MM, Marsh JW, Harrison LH Global epidemiology of capsular group W meningococcal disease (1970–2015): multifocal emergence and persistence of hypervirulent sequence type (ST)-11 clonal complex. Vaccine 2016; 34:1515–23. 26876439 10.1016/j.vaccine.2016.02.014

[2] Borrow R, Efron AM, Hill DMC, et al Genomic resolution of an aggressive, widespread, diverse and expanding meningococcal serogroup B, C and W lineage. J Infect 2015; 71:544–52. 26226598 10.1016/j.jinf.2015.07.007 PMC4635312

[3] Campbell H, Parikh SR, Borrow R, Kaczmarski E, Ramsay ME, Ladhani SN Presentation with gastrointestinal symptoms and high case fatality associated with group W meningococcal disease (MenW) in teenagers, England, July 2015 to January 2016. Euro Surveill 2016; 21:30175. 10.2807/1560-7917.ES.2016.21.12.30175 27035055

[4] Lucidarme J, Scott KJ, Ure R, et al An international invasive meningococcal disease outbreak due to a novel and rapidly expanding serogroup W strain, Scotland and Sweden, July to August 2015. Euro Surveill 2016; 21:30395. 27918265 10.2807/1560-7917.ES.2016.21.45.30395 PMC5144941

[5] Oldfield NJ, Green LR, Parkhill J, Bayliss CD, Turner DPJ Limited impact of adolescent meningococcal ACWY vaccination on *Neisseria meningitidis* serogroup W carriage in university students. J Infect Dis 2018; 217:608–16. 29155998 10.1093/infdis/jix596 PMC5853931

[6] Christensen H, May M, Bowen L, Hickman M, Trotter CL Meningococcal carriage by age: a systematic review and meta-analysis. Lancet Infect Dis 2010; 10:853–61. 21075057 10.1016/S1473-3099(10)70251-6

[7] Ala’Aldeen DAA, Oldfield NJ, Bidmos FA, et al Carriage of meningococci by university students, United Kingdom. Emerg Infect Dis 2011; 17:1761–3. 10.3201/eid1709.101762 PMC3322062 21888817

[8] Klughammer J, Dittrich M, Blom J, et al Comparative genome sequencing reveals within-host genetic changes in *Neisseria meningitidis* during invasive disease. PLoS One 2017; 12:e0169892. 28081260 10.1371/journal.pone.0169892 PMC5231331

[9] Lees JA, Oggioni MR, Kremer PHC, et al Large scale genomic analysis shows no evidence for pathogen adaptation between the blood and cerebrospinal fluid niches during bacterial meningitis. Microb Genomics 2016; 3:e000103. 10.1099/mgen.0.000103 PMC5361624 28348877

[10] Moxon ER, Bayliss CD, Hood DW Bacterial contingency loci: the role of simple sequence DNA repeats in bacterial adaptation. Annu Rev Genet 2006 40:307–33. 17094739 10.1146/annurev.genet.40.110405.090442

[11] Richardson AR, Stojiljkovic I Mismatch repair and the regulation of phase variation in *Neisseria meningitidis*. Mol Microbiol 2001; 40:645–55. 11359570 10.1046/j.1365-2958.2001.02408.x

[12] Bolle X De, Bayliss CD, Field D, et al The length of a tetranucleotide repeat tract in *Haemophilus influenzae* determines the phase variation rate of a gene with homology to type III DNA methyltransferases. Mol Microbiol 2000; 35:211–22. 10632891 10.1046/j.1365-2958.2000.01701.x

[13] Richardson AR, Yu Z, Popovic T, Stojiljkovic I Mutator clones of *Neisseria meningitidis* in epidemic serogroup A disease. Proc Natl Acad Sci U S A 2002; 99:6103–7. 11983903 10.1073/pnas.092568699 PMC122909

[14] Wanford JJ, Green LR, Aidley J, Bayliss CD Phasome analysis of pathogenic and commensal Neisseria species expands the known repertoire of phase variable genes, and highlights common adaptive strategies. PLoS One 2018; 13:e0196675. 29763438 10.1371/journal.pone.0196675 PMC5953494

[15] Alamro M, Bidmos FA, Chan H, et al Phase variation mediates reductions in expression of surface proteins during persistent meningococcal carriage. Infect Immun 2014; 82:2472–84. 24686058 10.1128/IAI.01521-14 PMC4019173

[16] Read RC, Baxter D, Chadwick DR, et al Effect of a quadrivalent meningococcal ACWY glycoconjugate or a serogroup B meningococcal vaccine on meningococcal carriage: an observer-blind, phase 3 randomised clinical trial. Lancet 2014; 384:2123–31. 25145775 10.1016/S0140-6736(14)60842-4

[17] Oldfield NJ, Cayrou C, AlJannat MAK, et al Rise in group W meningococcal carriage in university students, United Kingdom. Emerg Infect Dis 2017; 23:1009–11. 28518025 10.3201/eid2306.161768 PMC5443439

[18] Aidley J, Wanford JJ, Green LR, Sheppard SK, Bayliss CD PhasomeIt: an ‘omics’ approach to cataloguing the potential breadth of phase variation in the genus *Campylobacter*. Microb Genomics 2018; 4:e000228. 10.1099/mgen.0.000228 PMC6321876 30351264

[19] Green LR, Haigh RD, Bayliss CD Determination of repeat number and expression states of phase-variable loci through next generation sequencing and bioinformatic analysis. In: Seib K, Peak IR, eds. *Neisseria meningitidis*: methods and protocols. Springer, 2019. In press. 10.1007/978-1-4939-9202-7_5 30877670

[20] Al-Rubaiawi AA. Contribution of phase variation of Opa proteins to persistent carrige and immune evasion of *Neisseria meningitidis*. PhD Thesis, University of Leicester, Leicester, 2018 http://hdl.handle.net/2381/43068.

[21] Poolman JT, Kriz-Kuzemenska P, Ashton F, et al Serotypes and subtypes of *Neisseria meningitidis*: results of an international study comparing sensitivities and specificities of monoclonal antibodies. Clin Diagn Lab Immunol 1995; 2:69–72. 7719916 10.1128/cdli.2.1.69-72.1995 PMC170103

[22] Bayliss CD, Hoe JC, Makepeace K, Martin P, Hood DW, Moxon ER *Neisseria meningitidis* escape from the bactericidal activity of a monoclonal antibody is mediated by phase variation of *lgtG* and enhanced by a mutator phenotype. Infect Immun 2008; 76:5038–48. 18694967 10.1128/IAI.00395-08 PMC2573340

[23] Palmer ME, Lipsitch M, Moxon ER, Bayliss CD Broad conditions favor the evolution of phase-variable loci. MBio 2013; 4:e00430–12. 10.1128/mBio.00430-12 PMC3546556 23300246

[24] Capel E, Barnier JP, Zomer AL, et al Peripheral blood vessels are a niche for blood-borne meningococci. Virulence 2017; 8:1808–19. 29099305 10.1080/21505594.2017.1391446 PMC5810509

[25] Lucidarme J, Findlow J, Chan H, et al The distribution and ‘in vivo’ phase variation status of haemoglobin receptors in invasive meningococcal serogroup B disease: genotypic and phenotypic analysis. PLoS One 2013; 8:e76932. 24098814 10.1371/journal.pone.0076932 PMC3786947

[26] Jolley KA, Bray JE, Maiden MCJ Open-access bacterial population genomics: BIGSdb software, the PubMLST.org website and their applications. Wellcome Open Res 2018; 3:124. 30345391 10.12688/wellcomeopenres.14826.1 PMC6192448

