# Supplementary material for: Potentiation of Phase Variation in Multiple Outer-Membrane Proteins During Spread of the Hyperinvasive Neisseria meningitidis Serogroup W ST-11 Lineage
Source: J Infect Dis. 2019 May 23;220(7):1109–17. doi: 10.1093/infdis/jiz275 (PMC6735796; doi:10.1093/infdis/jiz275)
Supplement: jiz275_suppl_Supplementary_Data_File [file jiz275_suppl_supplementary_data_file.pdf]

upplementary Data File

This file contains the raw data on genomic and GeneScan analysis of the repeat numbers for the single-copy phase-variable OMPs encoding genes of UK invasive and carriage isolates of the MenW 11 clone plus tables showing the site of isolation (where known) for the invasive isolates and the selection of isolates used in the East Midlands and Random selection analyses.

These tables present the data in a range of data sets:- 2013_Invasive_2010_2018; Original_Invasive_2010_2018; Carriage 2010 (isolates from carriers during a 2010-2011 longitudinal carriage study performed in multiple UK universities); Carriage_2015_2016 (isolates from carriers from a 2015-2016 cross-sectional carriage study at the University of Nottingham).

Colour-coding of highlighted boxes in genomic data:- White, repeat numbers derived from assembled WGS genome sequences as found in Neisseria PubMLST; Orange, repeat numbers derived by analysis of read data mapped to a trap sequence (see text); Red, data missing due to poor assembly across the repeat tract; Green, .

Colour-coding of highlighted boxes in GeneScan data:- Red, OFF; Green, ON.

Other symbols and abbreviations:- x, missing data; D, December; M, March; S, September.

Gene Scan Data for Opa Genes in Carriage Isolates

Carriage Isolate	opaA	opaJ	opaB	opaD	Phasotype	OpaA-Repeat Number	OpaA-Expression State	OpaJ Repeat Number	OpaJ Expression State	OpaB Repeat Number	OpaB Expression State	OpaD Repeat Number	OpaD Expression State
**B003**	1	x	x	0	1xx0	8	1					22	0
**B005**	0	x	0	0	0x00	12	0			10	0	22	0
**B009**	0	1	1	0	0110	12	0	11	1	9	1	13	0
**B021**	1	1	0	0	1100	8	1	11	1	10	0	22	0
**B091**	0	1	x	x	01xx	10	0	8	1				
**B100**	0	x	x	x	0xxx	12	0						
**B110**	0	x	x	x	0xxx	12	0						
**B114**	0	x	x	0	0xx0	12	0					13	0
**B139**	x	0	1	0	x010			9	0	9	1	32	0
**B141**	x	0	0	0	x000			6	0	8	0	**25**	0
**B167**	0	1	0	0	0100	10	0	8	1	13	0	26	0
**B193**	0	0	x	x	00xx	12	0	7	0				
**B214**	0	x	x	x	0xxx	12	0						
**B222**	x	0	0	1	x001			16	0	7	0	15	1
**B227**	1	0	1	0	1010	9	1	16	0	9	1	28	0
**B240**	1	0	1	1	1011	8	1	12	0	9	1	24	1
**B243**	0	0	0	1	0001	7	0	10	0	7	0	18	1
**B251**	1	0	1	0	1010	9	1	10	0	9	1	22	0
**B253**	1	0	0	0	1000	8	1	10	0	10	0	22	0
**B256**	0	x	x	x	0xxx	7	0						
**B264**	0	0	0	0	0000	10	0	12	0	10	0	26	0
**B284**	0	0	0	0	0000	12	0	21	0	7	0	22	0
**B285**	1	0	0	x	100x	8	1	9	0	13	0	30	
**B295**	x	0	0	0	x000			9	0	10	0	26	0
**B346**	1	1	0	1	1101	14	1	14	1	11	0	12	1
**B366**	0	0	0	x	000x	7	0	12	0	7	0		

**B394**	1	1	0	0	1100	11	1	8	1	14	0	26	0
**B396**	1	0	0	1	1001	14	1	13	0	11	0	12	1
**R001**	0	0	0	0	0000	12	0	12	0	8	0	23	0
**R191**	1	0	1	0	1010	8	1	10	0	3	1	8	0
**R258**	0	1	0	0	0100	12	0	8	1	10	0	23	0
**R558**	1	1	x	x	11xx	11	1	8	1				
**R779**	x	0	1	0	x010			6	0	9	1	29	0
**R811**	0	0	x	0	00x0	10	0	16	0			19	0
**R825**	x	1	0	1	x101			8	1	8	0	12	1
**R838**	1	0	0	1	1001	8	1	10	0	10	0	33	1
**R852**	1	0	x	0	10x0	11	1	18	0			22	0
**R888**	1	x	x	x	1xxx	14	1						
**R889**	0	1	0	0	0100	12	0	14	1	8	0	18	0
**R920**	1	0	0	1	1001	11	1	15	0	8	0	15	1
**R941**	x	1	0	0	x100			17	1	10	0	25	0
**R949**	1	0	0	0	1000	8	1	22	0	10	0	26	0
**R958**	0	0	x	x	00xx	7	0	9	0				
**R980**	1	1	0	1	1101	11	1	8	1	11	0	28	1
**R990**	0	1	0	1	0101	13	0	17	1	11	0	12	1
**R998**	0	0	0	0	0000	10	0	7	0	8	0	29	0

Gene Scan Data for Opa Genes in Invasive Isolates

Disease Isolate	Epi Year	opa A	opa J	opaB	opaD	Phasotype	OpaA-Repeat Number	OpaA-Expression State	OpaJ Repeat Number	OpaJ-Expression State	OpaB Repeat Number	OpaB Expression State	OpaD Repeat Number	OpaD Expression State
M10 240514	07/2010-06/2011	1	0	0	0	1000	8	1	6	0	10	0	13	0
M10 240671	07/2010-06/2011		1	0	0	100			8	1	7	0	11	0
M10 240817	07/2010-06/2011	1		0	0	100	8	1			7	0	10	0
M10 240821	07/2010-06/2011	1	0	0	0	1000	5	1	9	0	8	0	13	0
M11 240035	07/2010-06/2011	1	0	0	0	1000	8	1	9	0	8	0	13	0
M11 240057	07/2010-06/2011	1	0	0	1	1001	8	1	7	0	10	0	15	1
M11 240067	07/2010-06/2011		0	0	1	001			7	0	10	0	15	1
M11 240099	07/2010-06/2011	0	1	0	0	0100	7	0	8	1	11	0	13	0
M11 240168	07/2010-06/2011	1	1	0	0	1100	8	1	8	1	8	0	13	0
M11 240305	07/2010-06/2011	1	0	0	0	1000	8	1	7	0	8	0	14	0
M11 240403	07/2010-06/2011	0	0	0	0	0000	7	0	**6**	0	10	0	**13**	0
M11 240417	07/2010-06/2011	0	0	1	1	0011	10	0	9	0	9	1	12	1
M11 240427	07/2010-06/2011	1	0	0	0	1000	8	1	7	0	7	0	11	0
M11 240726	07/2011-06/2012	1	0	0	0	1000	8	1	6	0	10	0	13	0
M11 240798	07/2011-06/2012	1	0	0	0	1000	8	1	6	0	8	0	11	0
M11 240802	07/2011-06/2012	1	1	0	0	1100	8	1	5	1	11	0	14	0
M11 240953	07/2011-06/2012	0	0	1	0	0010	7	0	9	0	9	1	20	0
M11 240975	07/2011-06/2012	1	0	0	0	1000	8	1	10	0	8	0	11	0
M12 240004	07/2011-06/2012	1	0	0	0	1000	8	1	7	0	10	0	11	0
M12 240067	07/2011-06/2012	1	1	0	0	1100	8	1	8	1	8	0	19	0

M12 240095	07/2011-06/2012	1	0	0	1	1001	8	1	6	0	11	0	12	1
M12 240125	07/2011-06/2012	1	0			10	8	1	7	0				
M12 240127	07/2011-06/2012	0	0			00	10	0	7	0				
M12 240133	07/2011-06/2012	0	1	0	0	0100	9	0	11	1	7	0	13	0
M12 240144	07/2011-06/2012	1	1				8	1	5	1				
M12 240156	07/2011-06/2012	0				0	10	0						
M12 240160	07/2011-06/2012	1				1	8	1						
M12 240240	07/2011-06/2012	1	0	0	1	1001	8	1	10	0	10	0	12	1
M12 240317	07/2011-06/2012		0	0		00			9	0	7	0		
M12 240324	07/2011-06/2012	1	0	0	0	1000	8	1	7	0	7	0	13	0
M12 240337	07/2012-06/2013	0	1	0	0	0100	9	0	11	1	7	0	8	0
M12 240640	07/2012-06/2013	1	0	0	0	1000	8	1	7	0	8	0	14	0
M12 240657	07/2012-06/2013	0	0			00	9	0	10	0				
M12 240663	07/2012-06/2013	1	0	0	0	1000	8	1	7	0	10	0	13	0
M12 240702	07/2012-06/2013		1	0	0	100			8	1	8	0	13	0
M12 240754	07/2012-06/2013	1	0	0	0	1000	8	1	9	0	16	0	16	0
M12 240774	07/2012-06/2013	0	0	0	0	0000	9	0	7	0	8	0	11	0
M12 240895	07/2012-06/2013	0	0	0	0	0000	6	0	12	0	8	0	10	0
M12 240898	07/2012-06/2013	0	0	0	1	0001	9	0	9	0	10	0	12	1
M13 240015	07/2012-06/2013	0	1	1	0	0110	7	0	14	1	12	1	17	0
M13 240025	07/2012-06/2013	1	0	0	0	1000	8	1	7	0	10	0	11	0
M13 240028	07/2012-06/2013	0	0	0	0	0000	9	0	6	0	8	0	11	0
M13 240246	07/2012-06/2013	1	0			10	8	1	9	0				

M13 240269	07/2012-06/2013	0	0	0	0	0000	7	0	13	0	7	0	16	0
M13 240283	07/2012-06/2013	0	0	1	0	0010	7	0	12	0	9	1	16	0
M13 240436	07/2012-06/2013	1		0	0	100	11	1			11	0	14	0
M13 240446	07/2012-06/2013	0	1			01	10	0	11	1				
M13 240491	07/2013-06/2014	1		0	1	101	8	1			13	0	15	1
M13 240531	07/2013-06/2014	0	1	1	1	0111	9	0	14	1	9	1	15	1
M13 240545	07/2013-06/2014	0	1	0	1	0101	7	0	17	1	11	0	12	1
M13 240553	07/2013-06/2014	1	1	0	0	1100	8	1	11	1	8	0	11	0
M13 240600	07/2013-06/2014	0	0	1	1	0011	7	0	13	0	9	1	12	1
M13 240672	07/2013-06/2014	1	0			10	8	1	13	0				
M13 240681	07/2013-06/2014	1	0	0	0	1000	11	1	18	0	8	0	13	0
M13 240705	07/2013-06/2014	0	1	0	1	0101	13	0	14	1	7	0	15	1
M13 240732	07/2013-06/2014	1	1	0	0	1100	11	1	8	1	10	0	19	0
M14 240026	07/2013-06/2014	1	0	0	0	1000	8	1	15	0	10	0	17	0
M14 240054	07/2013-06/2014	1	0	0	0	1000	8	1	12	0	13	0	16	0
M14 240072	07/2013-06/2014	0	1			01	7	0	8	1				
M14 240126	07/2013-06/2014	0	0	0	1	0001	7	0	12	0	7	0	12	1
M14 240142	07/2013-06/2014	1	0			10	8	1	13	0				
M14 240178	07/2013-06/2014	1	0			10	8	1	15	0				
M14 240180	07/2013-06/2014	1	0	0	0	1000	11	1	9	0	13	0	20	0
M14 240204	07/2013-06/2014	1	1	1	0	1110	8	1	17	1	9	1	13	0
M14 240257	07/2013-06/2014	1	1	0	0	1100	8	1	11	1	11	0	13	0
M14 240258	07/2013-06/2014	0	0	0	0	0000	7	0	9	0	8	0	11	0

M14 240283	07/2013-06/2014	1	0	0	0	1000	11	1	15	0	8	0	26	0
M14 240304	07/2013-06/2014	1		0	1	101	8	1			10	0	15	1
M14 240351	07/2013-06/2014	1	0	0	0	1000	8	1	13	0	8	0	11	0
M14 240389	07/2014-06/2015	1	1	0	0	1100	11	1	14	1	13	0	14	0
M14 240448	07/2014-06/2015	0	0	1	0	0010	6	0	16	0	12	1	13	0
M14 240468	07/2014-06/2015	0	1	0	1	0101	6	0	14	1	10	0	10	1
M14 240478	07/2014-06/2015	0	0	1	0	0010	18	0	12	0	12	1	14	0
M14 240485	07/2014-06/2015	0				0	6	0						
M14 240495	07/2014-06/2015	1	0	0	1	1001	11	1	15	0	10	0	12	1
M14 240496	07/2014-06/2015	1	0	0	0	1000	8	1	10	0	11	0	17	0
M14 240500	07/2014-06/2015	0	1	0	0	0100	9	0	14	1	8	0	23	0
M14 240511	07/2014-06/2015	0	0	0	1	0001	7	0	6	0	8	0	18	1
M14 240517	07/2014-06/2015	1	0			10	14	1	15	0				
M14 240524	07/2014-06/2015	1	1	0	0	1100	8	1	17	1	11	0	22	0
M14 240532	07/2014-06/2015	1	1	1	1	1111	8	1	14	1	9	1	18	1
M14 240550	07/2014-06/2015	1	1			11	11	1	11	1				
M14 240555	07/2014-06/2015	1	0	1	0	1010	11	1	10	0	12	1	14	0
M14 240556	07/2014-06/2015	0	0	0	0	0000	10	0	13	0	16	0	17	0
M14 240562	07/2014-06/2015	1	0	0	0	1000	11	1	9	0	7	0	11	0
M14 240573	07/2014-06/2015	0	0	0	0	0000	7	0	10	0	8	0	22	0
M14 240581	07/2014-06/2015	1		0	0	100	8	1			8	0	11	0
M14 240587	07/2014-06/2015	1	0	0	1	1001	8	1	10	0	11	0	21	1
M15 240871	07/2015-06/2016		0	0	1	001			7	0	10	0	15	1

M15 240873	07/2015-06/2016	0	1	0	0	0100	11	1	11	0	16	0
M15 240879	07/2015-06/2016	0	1	0	1	0101	14	1	11	0	12	1
M15 240888	07/2015-06/2016	0	0	0	0	0000	13	0	8	0	16	0
M15 240900	07/2015-06/2016			0	0	00			11	0	22	0
M15 240905	07/2015-06/2016	1	1	0	0	1100	11	1	8	0	22	0
M15 240937	07/2015-06/2016	0	0	0	0	0000	6	0	8	0	14	0
M15 240946	07/2015-06/2016	1	1	0	1	1101	8	1	8	0	18	1
M15 240952	07/2015-06/2016	0	0	0	1	0001	13	0	8	0	18	1
M15 240968	07/2015-06/2016	1	0	1		101	16	0	9	1		
M15 240978	07/2015-06/2016	1	0	1	0	1010	12	0	9	1	16	0
M15 240981	07/2015-06/2016	1	1	1	0	1110	11	1	9	1	11	0
M15 240983	07/2015-06/2016	0	0	0	0	0000	15	0	10	0	17	0
M15 240985	07/2015-06/2016	0	0	0	0	0000	15	0	11	0	26	0
M15 240995	07/2015-06/2016		0	0	0	000	9	0	8	0	10	0
M16 240008	07/2015-06/2016	1	1			11	11	1				
M16 240010	07/2015-06/2016	1	0	0	0	1000	6	0	7	0	11	0
M16 240015	07/2015-06/2016	1	0	0	0	1000	25	0	13	0	17	0
M16 240022	07/2015-06/2016	1	0	0	0	1000	18	0	13	0	26	0
M16 240024	07/2015-06/2016	0	1	1	0	0110	23	1	9	1	14	0
M16 240027	07/2015-06/2016	1	0	1	1	1011	13	0	9	1	18	1
M16 240039	07/2015-06/2016	1	0			10	18	0				
M16 240040	07/2015-06/2016	1	1			11	8	1				
M16 240041	07/2015-06/2016		0			0	22	0				

M16 240047	07/2015-06/2016		0			0	15	0				
M16 240052	07/2015-06/2016	1	0			10	7	0				
M16 240055	07/2015-06/2016		0	0	1	001	7	0	10	0	12	1
M16 240066	07/2015-06/2016		0			0	9	0				
M16 240072	07/2015-06/2016		1			1	8	1				
M16 240073	07/2015-06/2016	1	0	0	0	1000	7	0	8	0	27	0

Repeat Numbers for MenW cc11 2015-2016 Carriage Isolates Derived from Genome Sequence Data

Isolate	ENA Accession	BIGSDB ID	Date	Hall	Sequence Type (ST)	feta {NEIS1963} (Poly C)	fetA pro	nadA {NEIS1969} (5'-TAA A)	porA {NEIS1364} (Poly G)	porA pro	opcA {NEIS2198} (Poly C)	hpuA {NEIS1946} (Poly G)	nalP {NEIS1943} (Poly C)	mspA {NEIS1974} (Poly C)	hmbR (Poly G)	pilC1 {NEIS0371} (polyG)	pilC2 {NEIS0033} (polyG)	fetA	nadA	porA	hpuA	nalP	mspA	hmbR	pilC1	pilC2
**B003**	ERR2139023	51145	D	C	11	11	18	15	10	18	NS	10	10	9	10	10	11	1	0	2	1	1	1	0	1	0
**B005**	ERR2139315	54403	D	C	11	11	18	15	9	17	NS	10	10	9	10	10	12	1	0	0	1	1	1	0	1	1
**B009**	ERR2139024	51146	D	B	11	6	16	12	10	18	NS	9	10	11	9	13	8	2	0	2	0	1	0	1	1	0
**B018**	ERR2139317	54405	D	C	11	11	18	12	12	20	NS	9	10	8	10	10	10	1	0	1	0	1	0	0	1	0
**B021**	ERR2139025	51147	D	C	11	9	16	15	10	18	NS	9	10	9	10	13	11	2	0	2	0	1	1	0	1	0
**B064**	ERR2139324	54412	D	F	11	6	16	15	10	18	NS	10	10	11	9	13	12	2	0	2	1	1	0	1	1	1
**B091**	ERR2139026	51148	D	F	11	6	16	14	10	18	NS	10	11	10	8	13	10	2	2	2	1	0	0	0	1	0
**B103**	ERR2139333	54421	D	F	11	6	16	12	10	18	NS	12	11	11	9	13	8	2	0	2	0	0	0	1	1	0
**B110**	ERR2139028	51150	D	G	10651	6	16	14	7	16	NS	10	10	11	9	12	9	2	2	1	1	1	0	1	0	1
**B114**	ERR2139334	54422	D	F	11	6	16	14	10	18	NS	9	10	11	9	13	9	2	2	2	0	1	0	1	1	1
**B135**	ERR2139029	51151	M	E		6	16	12	9	17	NS	8	13	11	9	12	9	2	0	0	0	1	0	1	0	1
**B139**	ERR2139030	51152	M	E	11	6	16	12	8	17	NS	10	10	11	9	13	10	2	0	2	1	1	0	1	1	0
**B141**	ERR2139031	51153	M	E	11	6	16	12	8	17	NS	10	9	11	9	13	9	2	0	2	1	0	0	1	1	1
**B167**	ERR2139032	51154	M	E	11	6	16	12	10	18	NS	10	10	10	9	13	9	2	0	2	1	1	0	1	1	1
**B197**	ERR2139134	51250	M	E	11	6	16	15	10	18	NS	10	10	11	9	13	9	2	0	2	1	1	0	1	1	1
**B214**	ERR2139034	51156	M	A	11	6	16	9	10	18	NS	10	10	11	9	13	9	2	0	2	1	1	0	1	1	1
**B218**	ERR2139114	51258	M	A	11	6	16	15	8	16	NS	9	9	11	9	13	12	2	0	0	0	0	0	1	1	1
**B222**	ERR2139035	51157	M	A	11	6	16	12	10	18	NS	10	10	11	9	10	10	2	0	2	1	1	0	1	1	0
**B227**	ERR2139036	51158	M	A	11	6	16	15	9	17	NS	9	10	11	9	9	11	2	0	0	0	1	0	1	0	0
**B240**	ERR2139037	51159	M	C	11	12	19		11	19	NS	9	11	9	10	13	13	0	x	1	0	0	1	0	1	0
**B243**	ERR2139038	51160	M	C	11	12	19	15	8	16	NS	11	10	9	9	10	9	0	0	0	0	1	1	1	1	1
**B246**	ERR2139095	51266	M	C	11	6	16	12	10	18	NS	9	10	11	9	7	9	2	0	2	0	1	0	1	1	1

**B251**	ERR2139039	51161	M	C	11	12	19	15	8	16	NS	9	9	10	10	9	9	0	0	0	0	0	0	0	0	1
**B253**	ERR2139040	51162	M	C	11	11	18	15	10	18	NS	8	11	9	9	8	9	1	0	2	0	0	1	1	0	1
**B256**	ERR2139041	51163	M	C	11	13	20	15	12	20	NS	9	9	9	10	12	9	0	0	1	0	0	1	0	0	1
**B264**	ERR2139042	51164	M	C	11	11	18	14	9	17	NS	8	10	8	11	13		1	2	0	0	1	0	0	1	n/a
**B285**	ERR2139044	51166	M	C	11	6	16	11	9	17	NS	10	10	10	9	9	10	2	2	0	1	1	0	1	0	0
**B295**	ERR2139045	51167	M	C	11	6	16	9	9	17	NS	10	10	10	9	10	9	2	0	0	1	1	0	1	1	1
**B343**	ERR2139350	54439	M	D	11	15	22	14	8	16	NS	9	10	9	9	13	9	0	2	0	0	1	1	1	1	1
**B345**	ERR2139351	54440	M	D	11	13	20	15	10	18	NS	10	9	10	10	8	11	0	0	2	1	0	0	0	0	0
**B346**	ERR2139046	51168	M	F	11	6	16	12	10	18	NS	9	9	11	9	13	9	2	0	2	0	0	0	1	1	1
**B349**	ERR2139352	54441	M	F	11	6	16	12	10	18	NS	13	11	11	9	12	12	2	0	2	1	0	0	1	0	1
**B366**	ERR2139047	51169	M	F	11	11	18	12	10	18	NS	9	10	13	11	9	11	1	0	2	0	1	0	0	0	0
**B394**	ERR2139048	51170	M	F	11	6	16	12	10	18	NS	9	10	10	8	13	10	2	0	2	0	1	0	0	1	0
**B396**	ERR2139049	51171	M	F	11	6	16	12	10	18	NS	9	9	11	9	13	9	2	0	2	0	0	0	1	1	1
**R001**	ERR2139050	51172	S	D	11	6	16	12	10	18	NS	9	10	8	10	10	9	2	0	2	0	1	0	0	1	1
**R191**	ERR2139051	51173	S	D	11	6	16	9	7	16	NS	10	10	9	9		9	2	0	1	1	1	1	1	n/a	1
**R258**	ERR2139052	51174	S	F	11	6	16	12	10	18	NS	12	10	11	9	13	12	2	0	2	0	1	0	1	1	1
**R558**	ERR2139054	51176	S	G	10651	6	16	9	7	16	NS	9	11	10	9	11	9	2	0	1	0	0	0	1	0	1
**R575**	ERR2139275	51380	S	G	11	6	16	9	8	17	NS	10	10	8	10	12	9	2	0	2	1	1	0	0	0	1
**R779**	ERR2139055	51177	D	E	11	6	16	13	8	17	NS	10	9	10	9	14	10	2	1	2	1	0	0	1	0	0
**R811**	ERR2139056	51178	D	E	11	6	16	15	11	19	NS	10	10	10	9	10	13	2	0	1	1	1	0	1	1	0
**R825**	ERR2139057	51179	D	E	11	6	16	12	10	18	NS	10	10	11	9	13	9	2	0	2	1	1	0	1	1	1
**R838**	ERR2139058	51180	D	E	11	12	19	15	9	17	NS	9	9	9	10	10	11	0	0	0	0	0	1	0	1	0
**R852**	ERR2139059	51181	D	A	11	6	16	12	10	18	NS	9	10	11	9	13	12	2	0	2	0	1	0	1	1	1
**R888**	ERR2139060	51182	D	A	11	6	16	13	8	16	NS	9	9	11	9	13	10	2	1	0	0	0	0	1	1	0
**R889**	ERR2139061	51183	D	A	11	6	16	15	10	18	NS	9	10	11	9	13	10	2	0	2	0	1	0	1	1	0
**R920**	ERR2139062	51184	D	A	11	6	16	15	10	18	NS	10	10	11	9	13	9	2	0	2	1	1	0	1	1	1
**R941**	ERR2139063	51185	D	D	11	6	16	12	10	18	NS	10	10	11	9	13	13	2	0	2	1	1	0	1	1	0
**R949**	ERR2139064	51186	D	D	11	6	16	12	10	18	NS	10	10	11	9	13	13	2	0	2	1	1	0	1	1	0
**R958**	ERR2139065	51187	D	D	11	12	19	14	10	18	NS	10	11	9	10	9	13	0	2	2	1	0	1	0	0	0

**R980**	ERR2139066	51188	D	D	11	6	16	15	9	17	NS	10	10	10	9	13	9	2	0	0	1	1	0	1	1	1
**R990**	ERR2139067	51189	D	D	11	6	16	13	10	18	NS	10	9	11	9	13	9	2	1	2	1	0	0	1	1	1
**R998**	ERR2139068	51190	D	B	11	6	16	10	7	16	NS	10	10	11	9	13	8	2	1	1	1	1	0	1	1	0

Repeat Numbers for MenW cc11 2010-2011 Carriage Isolates Derived from Genome Sequence Data

Isolate	Accession	BIGSDB ID	Epidemiological Year	Month	Sequence Type (ST)	fetA (Poly C)	fetA pro	nadA (5'-TAAA)	porA (Poly G)	porA pro	hpuA (Poly G)	nalP (Poly C)	mspA (Poly C)	hmbR (Poly G)	pilC1 (poly G)	pilC2 (poly G)	fetA	nadA	porA	hpuA	nalP	mspA	hmbR	pilC1	pilC2
10117v2	ERR346730	26919	07/2010-06/2011	1	11	6	16	10	7	16	7	11	9	10	12	8	2	1	1	1	0	1	0	0	0
10117v3	ERR346731	26920	07/2010-06/2011	2	11	6	16	10	8	17	8	11	9	9	12	9	2	1	2	0	0	1	1	0	1
10117v4	ERR346732	26921	07/2010-06/2011	4	11	6	16	9	8	17	10	11	10	9	9	8	2	0	2	1	0	0	1	0	0
10507v4	ERR346736	26925	07/2010-06/2011	4	11	6	16	9	7	16	10	10	11	10	12	10	2	0	1	1	1	0	0	0	0
10507v5	ERR346737	26926	07/2010-06/2011	6	11	6	16	12	7	16	9	10	11	10	13	9	2	0	1	0	1	0	0	1	1
40118v3	ERR346742	26931	07/2010-06/2011	2	11	6	16	10	8	17	9	9	11	9	12	9	2	1	2	0	0	0	1	0	1
40118v4	ERR346743	26932	07/2010-06/2011	4	11	6	16	9	8	17	9	11	11	9	13	9	2	0	2	0	0	0	1	1	1
40161v1	ERR346744	26933	07/2010-06/2011	0	11	6	16	9	8	17	10	10	11	9	14	9	2	0	2	1	1	0	1	0	1
40161v2	ERR346745	26934	07/2010-06/2011	1	11	6	16	12	7	16	11	10	11	9	12	9	2	0	1	0	1	0	1	0	1
40161v3	ERR346746	26935	07/2010-06/2011	2	11	6	16	12	7	16	10	10	11	9	12	9	2	0	1	1	1	0	1	0	1
40161v4	ERR346747	26936	07/2010-06/2011	4	11	6	16	13	7	16	11	10	11	9	12	9	2	1	1	0	1	0	1	0	1
40161v5	ERR346748	26937	07/2010-06/2011	6	11	6	16	15	7	16	9	10	11	9	12	9	2	0	1	0	1	0	1	0	1
70322v3	ERR346757	26946	07/2010-06/2011	2	11	6	16	15	7	16	9	11	11	10	13	9	2	0	1	0	0	0	0	1	1
70322v4	ERR346758	26947	07/2010-06/2011	4	11	6	16	15	7	16	9	11	11	10	13	9	2	0	1	0	0	0	0	1	1
70322v5	ERR346759	26948	07/2010-06/2011	6	11	6	16	15	7	16	9	11	11	10	13	9	2	0	1	0	0	0	0	1	1
80046v4b	ERR346760	26949	07/2010-06/2011	4	10284	6	16	11	7	16	9	11	11	9	13		2	2	1	0	0	0	1	1	n/a
80046v5	ERR349238	26950	07/2010-06/2011	6	10284	6	16	13	7	16	10	11	11	9	13		2	1	1	1	0	0	1	1	n/a
80046v6	ERR349239	26951	07/2010-06/2011	12	10284	6	16	11	7	16	8	11	11	9	13		2	2	1	0	0	0	1	1	n/a
80164v1	ERR349240	26952	07/2010-06/2011	0	11	6	16	8	7	16	10	10	11	9	13	10	2	2	1	1	1	0	1	1	0
80164v2	ERR349241	26953	07/2010-06/2011	1	11	6	16	9	7	16	10	10	11	9	13	10	2	0	1	1	1	0	1	1	0
90021v3	ERR349245	26957	07/2010-06/2011	2	11	6	16	10	7	16	10	10	11	9	13		2	1	1	1	1	0	1	1	n/a
90021v4	ERR349246	26958	07/2010-06/2011	4	11	6	16	10	7	16	10	10	11	9	13		2	1	1	1	1	0	1	1	n/a
90022v2	ERR349247	26959	07/2010-06/2011	1	11	6	16	12	7	16	9	10	11	9	13	9	2	0	1	0	1	0	1	1	1
90022v3	ERR349248	26960	07/2010-06/2011	2	11	6	16	12	7	16	10	10	11	9	12	9	2	0	1	1	1	0	1	0	1

90022v4	ERR349249	26961	07/2010-06/2011	4	11	6	16	12	9	18	9	10	8	9	9	9	2	0	2	0	1	0	1	0	1
90034v4	ERR349251	26963	07/2010-06/2011	4	11	6	16	12	8	17	10	10	11	9	13	9	2	0	2	1	1	0	1	1	1
90034v6	ERR349252	26964	07/2010-06/2011	12	11	6	16	12	7	16	10	10	11	10	12	9	2	0	1	1	1	0	0	0	1
90058v2	ERR349253	26965	07/2010-06/2011	1	11	6	16	9	7	16	10	11	11	9	12		2	0	1	1	0	0	1	0	n/a
90058v3	ERR349254	26966	07/2010-06/2011	2	11	6	16	10	7	16	10	11	11	9	13		2	1	1	1	0	0	1	1	n/a
90058v4	ERR349255	26967	07/2010-06/2011	4	11	6	16	9	7	16	9	11	11	9	13		2	0	1	0	0	0	1	1	n/a
90075v2	ERR349257	26997	07/2010-06/2011	1	11	6	16	8	7	16	10	11	11	9	13		2	2	1	1	0	0	1	1	n/a
90075v3	ERR349258	26969	07/2010-06/2011	2	11	6	16	8	7	16	10	11	11	9	13		2	2	1	1	0	0	1	1	n/a
90092v1	ERR349259	26970	07/2010-06/2011	0	11	6	16	11	7	16	11	10	11	9	14	9	2	2	1	0	1	0	1	0	1
90092v2	ERR349260	26971	07/2010-06/2011	1	11	6	16	10	8	17	10	10	11	9	12	9	2	1	2	1	1	0	1	0	1
90092v5	ERR349261	26972	07/2010-06/2011	6	11	6	16	9	8	17	10	10	11	9	16	9	2	0	2	1	1	0	1	1	1
90092v6	ERR351638	26973	07/2010-06/2011	12	11	6	16	9	8	17	10	10	11	9		9	2	0	2	1	1	0	1	n/a	1
90102v3	ERR351639	26974	07/2010-06/2011	2	11	6	16	11	8	17	10	10	11	11	12	9	2	2	2	1	1	0	0	0	1
90102v4	ERR351642	26975	07/2010-06/2011	4	11	6	16	11	7	16	10	10	11	11		9	2	2	1	1	1	0	0	n/a	1
90111v4	ERR351643	26978	07/2010-06/2011	4	11	6	16	12	7	16	9	10	11	9	11	9	2	0	1	0	1	0	1	0	1
90111v5	ERR351644	26979	07/2010-06/2011	6	11	6	16	12	7	16	9	10	11	9	11	9	2	0	1	0	1	0	1	0	1
90119v2	ERR351645	26980	07/2010-06/2011	1	11	6	16	15	7	16	10	9	8	9	9	9	2	0	1	1	0	0	1	0	1
90119v3	ERR351646	26981	07/2010-06/2011	2	11	6	16	12	7	16	9	9	8	10	11	9	2	0	1	0	0	0	0	0	1
90119v4	ERR351647	26982	07/2010-06/2011	4	11	6	16	15	7	16	10	9	7	11	9	9	2	0	1	1	0	0	0	0	1
90119v5	ERR351653	26983	07/2010-06/2011	6	11	6	16	15	7	16	8	8	7	14	9	9	2	0	1	0	0	0	0	0	1
90136v3	ERR351654	26989	07/2010-06/2011	2	11	6	16	9	7	16	10	11	11	9	12		2	0	1	1	0	0	1	0	n/a
90136v4	ERR351656	26990	07/2010-06/2011	4	11	6	16	9	7	16	10	11	11	9	12		2	0	1	1	0	0	1	0	n/a
90142v1	ERR351661	26992	07/2010-06/2011	0	11	6	16	9	7	16	10	9	11	9	12	9	2	0	1	1	0	0	1	0	1

Repeat Numbers for Original-Strain MenW cc11 Invasive Isolates Derived from Genome Sequence Data

Isolate	BIGSDB ID	Sequence Type (ST)	fetA (Poly C)	fetA pro	nadA (5'-TAA A)	porA	porA pro	opcA (Poly C)	hpuA (Poly G)	nalP (Poly C)	mspA (Poly C)	hmbR (Poly G)	pilC1 (Poly G)	pilC2 (Poly G)	fetA	nadA	porA	hpuA	nalP	mspA	hmbR	pilC1	pilC2
M10 240514	19968	11	6	16	12	9	17	NS	9	10	11	9	11	9	2	0	0	0	1	0	1	0	1
M10 240671	20057	11	6	16	17	7	16	NS	11	10	11	7	12	8	2	0	1	0	1	0	0	0	0
M10 240817	20154	11	6	16	12	9	18	NS	9	11	8	10	9	8	2	0	2	0	0	0	0	0	0
M10 240821	20158	11	6	16	15	7	16	NS	11	10	11	9	12	10	2	0	1	0	1	0	1	0	0
M11 240035	20196	11	6	16	8	7	16	NS	10	11	11	9	13		2	2	1	1	0	0	1	1	n/a
M11 240057	20216	11	6	16	12	7	16	NS	10	11	11	9	12	9	2	0	1	1	0	0	1	0	1
M11 240067	20226	11	6	16	12	7	16	NS	10	11	11	9	12	10	2	0	1	1	0	0	1	0	0
M11 240099	20247	11	6	16	9	7	16	NS	10	10	10	9	13	13	2	0	1	1	1	0	1	1	0
M11 240168	20288	11	6	16	12	7	16	NS	10	10	11	9	11	9	2	0	1	1	1	0	1	0	1
M11 240305	20368	11	6	16	9	7	16	NS	10	10	11	9	13		2	0	1	1	1	0	1	1	n/a
M11 240389	20424	11	6	16	9	11	19	NS	9	9	10	9	12	9	2	0	1	0	0	0	1	0	1
M11 240403	20436	11	6	16	14	7	16	NS	10	10	11	9		9	2	2	1	1	1	0	1	n/a	1
M11 240417	20444	11	6	16	9	7	16	NS	9	11	10	9	14		2	0	1	0	0	0	1	0	n/a
M11 240427	20449	11	6	16	12	7	16	NS	10	11	11	9		9	2	0	1	1	0	0	1	n/a	1
M12 240156	20460	11	6	16	12	10	18	NS	10	10	10	10	12	12	2	0	2	1	1	0	0	0	1
M12 240160	20461	11	6	16	9	7	16	NS	10	10	10	10	20	13	2	0	1	1	1	0	0	0	0
M11 240486	21123	1860	6	16	9	7	16	NS	10	10	11	9	12	9	2	0	0	1	1	0	1	0	1
M11 240726	21163	11	6	16	9	7	16	NS	10	10	11	9		12	2	0	1	1	1	0	1	n/a	1
M11 240798	21203	11	6	16	9	13	19	NS	10	10	8	10	9	10	2	0	1	1	1	0	0	0	0
M11 240802	21206	11	6	16	17	7	16	NS	10	10	11	10	15	10	2	0	1	1	1	0	0	0	0
M11 240953	21214	11	6	16	8	10	19	NS	10	10	11	10	13	10	2	2	1	1	1	0	0	1	0
M11 240975	21216	11	6	16	9	7	16	NS	9	10	11	9	12	10	2	0	1	0	1	0	1	0	0
M12 240004	21288	11	6	16	9	7	16	NS	9	10	11	9	13	8	2	0	1	0	1	0	1	1	0
M12 240016	21298	11	6	16	9	7	16	NS	10	10	11	10	9	10	2	0	1	1	1	0	0	0	0
M12 240021	21302	11	6	16	8	7	16	NS	10	10	11	10	11	10	2	2	1	1	1	0	0	0	0

M12 240027	21305	11	6	16	8	7	16	NS	10	10	11	8	12	9	2	2	1	1	1	0	0	0	1
M12 240067	21334	11	6	16	11	7	16	NS	9	10	11	9	12		2	2	1	0	1	0	1	0	n/a
M12 240095	21354	11	6	16	9	7	16	NS	10	10	11	9	13	9	2	0	1	1	1	0	1	1	1
M12 240125	21375	11	6	16	9	7	16	NS	9	10	11	9	20	10	2	0	1	0	1	0	1	0	0
M12 240127	21377	11	6	16	9	7	16	NS	9	10	10	9	12	9	2	0	1	0	1	0	1	0	1
M12 240133	21381	11	6	16	10	6	15	NS	10	10	11	9		9	2	1	0	1	1	0	1	n/a	1
M12 240144	21386	10284	6	16	12	7	16	NS	10	11	11	9	12	10	2	0	1	1	0	0	1	0	0
M12 240240	21446	11	6	16	13	7	16	NS	10	10	11	9	12	11	2	1	1	1	1	0	1	0	0
M12 240317	21492	11	6	16	12	7	16	NS	9	9	10	9	11	9	2	0	1	0	0	0	1	0	1
M12 240324	21499	11	6	16	15	7	16	NS	10	8	11	9	11		2	0	1	1	0	0	1	0	n/a
M12 240337	28114	11	6	16	12	11	17	NS	10	10	11	9	10	9	2	0	2	1	1	0	1	1	1
M12 240640	28115	11	6	16	10	7	16	NS	10	10	11	10	12	10	2	1	1	1	1	0	0	0	0
M12 240657	28116	11	6	16	8	7	16	NS	9	10	10	9		9	2	2	1	0	1	0	1	n/a	1
M12 240663	28117	11	6	16	9	11	17	NS	10	11	11	9	15	10	2	0	2	1	0	0	1	0	0
M12 240702	28119	11	9	16	9	7	16	NS	10	10	10	9	12	10	2	0	1	1	1	0	1	0	0
M12 240751	28121	10651	6	16	7	6	15	NS	11	11	11	9		9	2	1	0	0	0	0	1	n/a	1
M12 240754	28122	11	6	16	9	8	17	NS	7	10	9	9		9	2	0	2	1	1	1	1	n/a	1
M12 240774	28125	11	6	16	15	7	16	NS	10	10	12	9	13		2	0	1	1	1	1	1	1	n/a
M12 240826	28128	11	6	16	9	7	16	NS	10	10	12	9		10	2	0	1	1	1	1	1	n/a	0
M12 240895	28131	11	6	16	9	7	16	NS	10	10	10	9	12	9	2	0	1	1	1	0	1	0	1
M12 240898	28132	11	6	16	17	7	16	NS	8	9	8	9	9	8	2	0	1	0	0	0	1	0	0
M13 240025	28135	11	6	16	10	11	20	NS	10	7	8	11	12	9	2	1	1	1	1	0	0	0	1
M13 240028	28136	11	6	16	8	7	16	NS	10	10	9	11	9	10	2	2	1	1	1	1	0	0	0
M13 240056	28137	11	6	16	9	7	16	NS	10	10	15	10		10	2	0	1	1	1	1	0	n/a	0
M13 240066	28138	11	6	16	16	7	16	NS	9	10	11	9	11	10	2	1	1	0	1	0	1	0	0
M13 240077	28139	10651	6	16	10	7	16	NS	10	10	11	9	8	9	2	1	1	1	1	0	1	0	1
M13 240109	28141	11	6	16	9	7	16	NS	8	9	10	9	11		2	0	1	0	0	0	1	0	n/a
M13 240114	28142	11	6	16	10	7	16	NS	11	11	11	9	16	9	2	1	1	0	0	0	1	1	1
M13 240158	28143	11	6	16	10	7	16	NS	9	10	11	9	13	9	2	1	1	0	1	0	1	1	1

M13 240168	28144	11	6	16	12	7	16	NS	11	10	10	9		11	2	0	1	0	1	0	1	n/a	0
M13 240185	28146	10651	6	16	9	11	19	NS	10	11	11	9	12	9	2	0	1	1	0	0	1	0	1
M13 240223	28147	11	6	16	12	7	16	NS	10	9	11	9	12	10	2	0	0	1	0	0	1	0	0
M13 240225	28148	10651	6	16	9	11	19	NS	9	12	11	9	12	10	2	0	1	0	0	0	1	0	0
M13 240238	28149	11	6	16	10	7	16	NS	9	9	11	9	11	10	2	1	0	0	0	0	1	0	0
M13 240247	28151	11	6	16	12	10	18	NS	10	10	11	9		13	2	0	2	1	1	0	1	n/a	0
M13 240251	28152	11	6	16	9	7	16	NS	9	10	10	9		10	2	0	0	0	1	0	1	n/a	0
M13 240457	28158	11	6	16	9	17	23	NS	11	10	13	9	12	13	2	0	0	0	1	0	1	0	0
M13 240464	28159	11	6	16	12	7	16	NS	10	11	11	9	13	8	2	0	1	1	0	0	1	1	0
M13 240467	28161	11	6	16	6	7	16	NS	9	10	11	9	12	9	2	0	1	0	1	0	1	0	1
M13 240469	28162	11	6	16	9	12	20	NS	9	10	11	9	12		2	0	1	0	1	0	1	0	n/a
M13 240473	28163	11	6	16	12	7	16	NS	9	10	10	8	9	8	2	0	0	0	1	0	0	0	0
M13 240482	28164	11	6	16	8	7	16	NS	9	10	9	9	9	8	2	2	1	0	1	1	1	0	0
M13 240555	29723	11	6	16	8	8	17	NS	10	10	11	9	13	9	2	2	2	1	1	0	1	1	1
M13 240591	29724	11	6	16	9	7	16	NS	10	11	8	11	12	10	2	0	1	1	0	0	0	0	0
M13 240603	29725	11	6	16	12	7	16	NS	10	11	10	9	14	11	2	0	1	1	0	0	1	0	0
M13 240604	29726	10651	6	16	12	7	16	NS	11	10	11	9	12	9	2	0	1	0	1	0	1	0	1
M13 240558	29728	11	6	16	12	7	16	NS	10	9	10	9	12	8	2	0	1	1	0	0	1	0	0
M13 240594	29729	11	6	16	12	6	15	NS	11	11	10	9	13	9	2	0	0	0	0	0	1	1	1
M13 240583	29730	11161	6	16	12	7	16	NS	8	10	8	14	11	9	2	0	1	0	1	0	0	0	1
M13 240510	29731	11	6	16	9	7	16	NS	9	10	11	9	14	12	2	0	1	0	1	0	1	0	1
M13 240633	29732	11	6	16	9	7	16	NS	6	11	8	9	7	12	2	0	1	0	0	0	1	1	1
M13 240634	29733	11	6	16	12	9	17	NS	10	10	11	9	14	12	2	0	2	1	1	0	1	0	1
M13 240538	29734	11	6	16	15	7	16	NS	10	10	10	9	14	9	2	0	1	1	1	0	1	0	1
M13 240515	29735	11	6	16	12	7	16	NS	10	10	12	10	12	13	2	0	1	1	1	1	0	0	0
M13 240635	30136	11	6	16	6	6	15	NS	10	9	10	9	14	10	2	0	0	1	0	0	1	0	0
M13 240637	30137	11	6	16	9	8	17	NS	9	11	8	10	12	10	2	0	2	0	0	0	0	0	0
M13 240649	30138	11	6	16	9	10	18	NS	9	10	8	11	9	10	2	0	2	0	1	0	0	0	0
M13 240655	30139	11	6	16	7	7	16	NS	10	10	11	9	14	9	2	1	0	1	1	0	1	0	1
M13 240657	30140	11	6	16	15	7	16	NS	9	10	11	9	13		2	0	1	0	1	0	1	1	n/a

M13 240664	30141	11	6	16	9	7	16	NS	10	11	11	10	13	10	2	0	1	1	0	0	0	1	0
M13 240667	30143	11	6	16	9	7	16	NS	10	10	12	9	12	10	2	0	1	1	1	1	1	0	0
M13 240668	30144	11	6	16	9	15	19	NS	10	10	12	9	12	10	2	0	1	1	1	1	1	0	0
M13 240678	30146	11	6	16	12	7	16	NS	8	10	9	9	9	10	2	0	1	0	1	1	1	0	0
M13 240680	30147	11	6	16	12	7	16	NS	10	10	10	9	13	10	2	0	1	1	1	0	1	1	0
M13 240724	30150	11	6	16	9	7	16	NS	10	10	11	9	10	10	2	0	1	1	1	0	1	1	0
M13 240726	30151	11	6	16	6	8	17	NS	9	10	11	9	12	9	2	0	2	0	1	0	1	0	1
M13 240730	30152	11	6	16	9	9	17	NS	7	10	8	11	9	11	2	0	2	1	1	0	0	0	0
M14 240001	30154	11	6	16	7	7	16	NS	11	10	10	9	13		2	1	0	0	1	0	1	1	n/a
M14 240002	30155	11	6	16	12	7	16	NS	9	11	11	9		10	2	0	1	0	0	0	1	n/a	0
M14 240007	30156	11	6	16	19	7	16	NS	10	10	11	11	14	11	2	0	1	1	1	0	0	0	0
M14 240013	30157	11	6	16	11	7	16	NS	9	11	11	9	13	10	2	2	1	0	0	0	1	1	0
M14 240019	30158	11	6	16	9	7	16	NS	9	10	11	9	14		2	0	1	0	1	0	1	0	n/a
M14 240022	30159	8621	6	16	9	10	18	NS	10	10	12	10	13	11	2	0	2	1	1	1	0	1	0
M14 240031	30162	11	6	16	7	9	18	NS	10	10	8	9	12	10	2	1	2	1	1	0	1	0	0
M14 240042	30163	11	6	16	12	12	20	NS	10	10	11	9	14		2	0	1	1	1	0	1	0	n/a
M14 240043	30164	11	6	16	20	7	16	NS	10	11	10	9	9	8	2	0	0	1	0	0	1	0	0
M14 240052	30165	11	6	16		7	16	NS	10	10	12	9	12		2	n/a	1	1	1	1	1	0	n/a
M14 240053	30166	10651	6	16	13	7	16	NS	9	10	11	9	12	10	2	1	1	0	1	0	1	0	0
M14 240082	30169	11	6	16	13	7	16	NS	10	10	10	9	13	9	2	1	1	1	1	0	1	1	1
M14 240447	31168	11	6	16	13	9	17	NS	10	10	11	9	12	9	2	1	2	1	1	0	1	0	1
12.7350.H	35371	11	6	16	15	7	16	NS	9	11	11	9	11	8	2	0	1	0	0	0	1	0	0
M14 240090	35581	11	6	16	9	7	16	NS	10	10	10	9	12	10	2	0	1	1	1	0	1	0	0
M14 240116	35598	11	6	16	6	7	16	NS	10	10	11	8	12	10	2	0	1	1	1	0	0	0	0
M14 240135	35614	11	6	16	12	7	16	NS	10	10	11	8	11	10	2	0	1	1	1	0	0	0	0
M14 240145	35621	11	6	16	12	7	16	NS	10	10	10	10	12	9	2	0	1	1	1	0	0	0	1
M14 240147	35622	11	6	16	9	7	16	NS	11	10	11	9	13		2	0	1	0	1	0	1	1	n/a
M14 240150	35624	11	6	16	9	6	15	NS	9	9	10	8	10	11	2	0	0	0	0	0	0	1	0
M14 240199	35635	11	6	16	13	10	18	NS	9	10	10	10		9	2	1	2	0	1	0	0	n/a	1
M14 240211	35643	11	6	16	9	7	16	NS	10	10	12	10	11		2	0	1	1	1	1	0	0	n/a

M14 240223	35651	11	6	16	15	7	16	NS	10	10	8	9	9	10	2	0	1	1	1	0	1	0	0
M14 240245	35664	11292	6	16	9	6	15	NS	9	10	10	9	11	10	2	0	0	0	1	0	1	0	0
M14 240253	35670	11	6	16	12	10	18	NS	10	10	11	10	13	9	2	0	2	1	1	0	0	1	1
M14 240278	35686	11	6	16	14	11	19	NS	9	10	11	9	10	10	2	2	1	0	1	0	1	1	0
M14 240279	35687	10651	6	16	11	9	17	NS	10	10	11	10	11	9	2	2	2	1	1	0	0	0	1
M14 240282	35689	11	6	16	15	8	17	NS	10	10	10	9	13	10	2	0	2	1	1	0	1	1	0
M14 240299	35700	10651	6	16	15			NS	11	12	11	9	12	9	2	0	n/a	0	0	0	1	0	1
M14 240337	35722	11	6	16	12	7	16	NS	9	10	12	10	11	10	2	0	1	0	1	1	0	0	0
M14 240343	35725	11	6	16	12	7	16	NS	9	11	11	9	11	9	2	0	1	0	0	0	1	0	1
M14 240346	35728	11	6	16	9	7	16	NS	9	9	8	9	9	11	2	0	1	0	0	0	1	0	0
M14 240391	35756	11	6	16	12	8	17	NS	10	10	11	9		9	2	0	0	1	1	0	1	n/a	1
M14 240406	35763	11	6	16	12	7	16	NS	9	10	11	10		9	2	0	1	0	1	0	0	n/a	1
M14 240463	35782	11	6	16	9	7	16	NS	9	10	11	9	13	9	2	0	0	0	1	0	1	1	1
M14 240470	35788	11	6	16	13	7	16	NS	11	10	10	9	12	13	2	1	1	0	1	0	1	0	0
M14 240474	35792	11	6	16	12	10	18	NS	10	10	11	10	12	11	2	0	2	1	1	0	0	0	0
M14 240482	35799	11	6	16	12	10	18	NS	9	10	10	9	11	9	2	0	2	0	1	0	1	0	1
M14 240486	35801	11	6	16	8	7	16	NS	9	10	10	10	9	8	2	2	0	0	1	0	0	0	0
M14 240492	35807	10651	6	16	12	6	15	NS	10	10	11	9	12	9	2	0	0	1	1	0	1	0	1
M14 240494	35808	11	6	16	12	10	18	NS	10	9	11	10	13	8	2	0	2	1	0	0	0	1	0
M14 240394	37670	11	6	16		10	18	NS	11	9	11	9	13	15	2	n/a	2	0	0	0	1	1	1
M14 240502	37673	10651	6	16	9	7	16	NS	9	10	9	9	11	8	2	0	1	0	1	1	1	0	0
M14 240504	37675	10651	6	16	12	7	16	NS	11	11	11	9	11	9	2	0	1	0	0	0	1	0	1
M14 240522	37690	11	6	16	15	7	16	NS	11	11	11	10	8	8	2	0	1	0	0	0	0	0	0
M14 240525	37693	11	6	16	13	9	17	NS	10	9	12	9	12	10	2	1	0	1	0	1	1	0	0
M14 240534	37701	11	6	16	12	8	17	NS	10	10	11	9	10	11	2	0	2	1	1	0	1	1	0
M14 240545	37708	11	6	16	12	7	16	NS	9	10	9	9		9	2	0	1	0	1	1	1	n/a	1
M14 240548	37710	11	6	16	10	9	17	NS	11	9	11	9	12	9	2	1	0	0	0	0	1	0	1
M14 240572	37730	11	6	16	12	7	16	NS	10	10	11	9	11	9	2	0	1	1	1	0	1	0	1
M14 240574	37732	11	6	16	9	9	17	NS	8	10	9	10	9	12	2	0	0	0	1	1	0	0	1
M14 240585	37738	10651	6	16		7	16	NS	10	10	10	8	11	8	2	n/a	1	1	1	0	0	0	0

M14 240593	37742	11	6	16	12	7	16	NS	10	10	11	9	12		2	0	1	1	1	0	1	0	n/a
M14 240601	37746	11867	6	16	9	9	17	NS	9	10	10	9	9	11	2	0	0	0	1	0	1	0	0
M14 240605	37749	10651	6	16	14	7	16	NS	11	12	12	10	13	11	2	2	1	0	0	1	0	1	0
M14 240649	37784	11	6	16	9	9	17	NS	10	10	11	10	12	8	2	0	0	1	1	0	0	0	0
M15 240010	37797	11	6	16	9	6	15	NS	10	10	10	10	12	6	2	0	0	1	1	0	0	0	1
M15 240015	37801	11	6	16	11	7	16	NS	9	10	8	9	13	10	2	2	1	0	1	0	1	1	0
M15 240021	37805	11	6	16	12	7	16	NS	10	10	10	10		11	2	0	1	1	1	0	0	n/a	0
M15 240022	37806	11	6	16	12	9	17	NS	10	10	10	10	12	9	2	0	2	1	1	0	0	0	1
M15 240027	37809	11	6	16	12	7	16	NS	10	10	11	9	10		2	0	1	1	1	0	1	1	n/a
M15 240029	37811	11	6	16	15	7	16	NS	10	10	11	9	7		2	0	1	1	1	0	1	1	n/a
M15 240041	37821	11	6	16	9	7	16	NS	10	10	9	10	9	11	2	0	0	1	1	1	0	0	0
M15 240048	37828	11	6	16	12	7	16	NS	8	11	8	15?	9	12	2	0	1	0	0	0	0	0	1
M15 240066	37845	11	6	16	12	7	16	NS	6	10	11	9	11	9	2	0	1	0	1	0	1	0	1
M15 240078	37854	11	6	16	8	7	16	NS	10	10	11	9	11	13	2	2	1	1	1	0	1	0	0
M15 240081	37857	11	6	16	12	7	16	NS	10	11	11	9		10	2	0	1	1	0	0	1	n/a	0
M15 240114	37881	11	6	16	9	8	17	NS	10	10	9	10		10	2	0	2	1	1	1	0	n/a	0
M15 240127	37890	11	6	16		9	17	NS	9	9	10	8	9	8	2	n/a	2	0	0	0	0	0	0
M15 240130	37893	11	6	16	13	7	16	NS	6	10	11	9	11	8	2	1	1	0	1	0	1	0	0
M15 240141	37902	10651	6	16	6	7	16	NS	10	10	11	9	14	10	2	0	1	1	1	0	1	0	0
M15 240144	37904	11	6	16	9	7	16	NS	10	10	11	10	13	9	2	0	0	1	1	0	0	1	1
M15 240151	37910	11	6	16	9	7	16	NS	10	10	10	10	12		2	0	1	1	1	0	0	0	n/a
M15 240158	37915	11	6	16	9	7	16	NS	11	10	10	9	16	16	2	0	1	0	1	0	1	1	0
M15 240172	37922	10651	6	16		7	16	NS	10	9	11	10	12	8	2	n/a	1	1	0	0	0	0	0
M15 240181	37930	11	6	16	11	11		NS	10	10	9	10	13	10	2	2	n/a	1	1	1	0	1	0
M15 240185	37933	11	6	16	9	7	16	NS	10	10	11	9	12	10	2	0	1	1	1	0	1	0	0
M15 240187	37934	11	6	16	12	7	16	NS	10	10	12	10	14	9	2	0	1	1	1	1	0	0	1
M15 240189	37936	11	6	16	9	7	16	NS	10	9	11	9	12	9	2	0	0	1	0	0	1	0	1
M15 240242	37977	11	6	16	9	7	16	NS	10	10	9	9	12	10	2	0	1	1	1	1	1	0	0
M15 240244	37978	11	6	16	12	11		NS	9	10	11	9	13	8	2	0	n/a	0	1	0	1	1	0
M15 240248	37981	11	6	16		9	17	NS	10	10	10	10	14	9	2	n/a	2	1	1	0	0	0	1

M15 240271	37995	11	6	16	12	7	16	NS	10	11	11	10		9	2	0	1	1	0	0	0	n/a	1
M15 240277	37998	11	6	16	9			NS	9	10	11	9	11	8	2	0	n/a	0	1	0	1	0	0
M15 240278	37999	11	6	16	12	7	16	NS	10	11	11	9	13	8	2	0	0	1	0	0	1	1	0
M15 240280	38001	10651	6	16	12	7	16	NS	10	10	11	10	13	9	2	0	1	1	1	0	0	1	1
M15 240282	38003	11	6	16	12	7	16	NS	10	10	10	10	11	11	2	0	0	1	1	0	0	0	0
M15 240284	38004	11	6	16	12	7	16	NS	10	10	11	10	12	8	2	0	1	1	1	0	0	0	0
M15 240310	38024	11	6	16	8	8	17	NS	6	10	11	9	12	10	2	2	2	0	1	0	1	0	0
M15 240326	38034	11	6	16		7	16	NS	10	10	10	9	9	9	2	n/a	1	1	1	0	1	0	1
M15 240334	38049	11	6	16	9	9		NS	10	10	10	9	12	12	2	0	n/a	1	1	0	1	0	1
M15 240375	38059	10651	6	16	12	9	17	NS	9	9	10	10	11	10	2	0	2	0	0	0	0	0	0
M15 240376	38060	11	6	16		7	16	NS	10	11	9	9	11	10	2	n/a	1	1	0	1	1	0	0
M15 240380	38063	11	6	16	12	9	17	NS	7	9	9	8	9	10	2	0	0	1	0	1	0	0	0
M15 240381	38064	11	6	16	9	7	16	NS	9	13	9	8	9	10	2	0	0	0	1	1	0	0	0
M15 240384	38067	11	6	16	12	6	15	NS	10	10	10	9	13	13	2	0	0	1	1	0	1	1	0
M15 240438	38076	11	6	16	12	7	16	NS	9	10	9	9	12	8	2	0	0	0	1	1	1	0	0
M15 240477	38096	11491	6	16	3	8	17	NS	10	10	10	9	10	8	2	0	2	1	1	0	1	1	0
15.8700914	38996	11	6	16	13	8	17	NS	9	10	11	10	11	10	2	1	2	0	1	0	0	0	0
15.8703957	39000	11	6	16	12	7	16	NS	10	10	11	9		12	2	0	1	1	1	0	1	n/a	1
15.8705087	39001	11	6	16	8	7	16	NS	8	10	8	10	11	17	2	2	1	0	1	0	0	0	0
M15 240638	39326	11	6	16	10	9	18	NS	9	10	9	8	9	10	2	1	2	0	1	1	0	0	0
M15 240640	39328	11	6	16	9	7	16	NS	10	10	10	10	11		2	0	1	1	1	0	0	0	n/a
M15 240657	39340	11	6	16	13	7	16	NS	10	10	12	10	12	11	2	1	1	1	1	1	0	0	0
M15 240662	39345	11	6	16	12	10	18	NS	10	9	11	9	12	10	2	0	2	1	0	0	1	0	0
M15 240664	39346	11	6	16	12	10	18	NS	10	12	11	9	11	10	2	0	2	1	0	0	1	0	0
M15 240730	39365	11	6	16	9	7	16	NS	9	10	11	9	12	8	2	0	1	0	1	0	1	0	0
M15 240746	39373	11	6	16	12			NS	10	10	11	10		10	2	0	n/a	1	1	0	0	n/a	0
M15 240762	39385	11	6	16	9	6	15	NS	9		11	9	12		2	0	0	0	n/a	0	1	0	n/a
M15 240778	39395	11	6	16	15	7	16	NS	9	10	11	9	11	11	2	0	1	0	1	0	1	0	0
M15 240779	39396	11	6	16	10	8	16	NS	10	10	12	10	12	12	2	1	1	1	1	1	0	0	1
M15 240782	39398	11	6	16	12	7	16	NS	11	10	11	9	12	9	2	0	0	0	1	0	1	0	1

M15 240788	39404	11	6	16	12	7	16	NS	10	10	10	10	12	11	2	0	1	1	1	0	0	0	0
M15 240794	39408	11	6	16	12	11	20	NS	9	10	8	9	9	10	2	0	1	0	1	0	1	0	0
M15 240797	39411	11	6	16	17	7	16	NS	10	10	9	13	9	9	2	0	1	1	1	1	0	0	1
M15 240798	39412	11	6	16	12	7	16	NS	10	10	12	9	12	9	2	0	1	1	1	1	1	0	1
M15 240826	39427	11	6	16	9	7	16	NS	9	10	11	9		10	2	0	1	0	1	0	1	n/a	0
M15 240848	39438	11	6	16	12	10	16	NS	10	10	11	9	13	9	2	0	1	1	1	0	1	1	1
M15 240852	40322	11	6	16	9			NS			10	9		9	2	0	n/a	n/a	n/a	0	1	n/a	1
M15 240753	41451	11	6	16	15	7	16	NS	9	10	10	9	11	12	2	0	1	0	1	0	1	0	1
M15 240856	41455	11	6	16	12	7	16	NS	10	10	11	9		10	2	0	1	1	1	0	1	n/a	0
M15 240864	41462	11	6	16	9	7	16	NS	9	11	10	9	9	9	2	0	1	0	0	0	1	0	1
M15 240876	41470	11	6	16	10	10	19	NS	9	10	10	13	10	11	2	1	1	0	1	0	0	1	0
M15 240885	41478	11	6	16	15	10	16	NS	10	10	11	9		12	2	0	1	1	1	0	1	n/a	1
M15 240896	41486	11	6	16	13	8	17	NS	10	10	9	8	9	8	2	1	2	1	1	1	0	0	0
M15 240918	41501	11	6	16	9	7	16	NS	10	10	11	9	11	10	2	0	1	1	1	0	1	0	0
M15 240919	41502	11	6	16	12	11	19	NS	10	9	8	9	9	8	2	0	1	1	0	0	1	0	0
M15 240936	41515	11	6	16	6	9	17	NS	14?	10	9	9	10	8	2	0	0	0	1	1	1	1	0
M15 240940	41519	11	6	16	12	10	18	NS	10	10	10	11	12	11	2	0	2	1	1	0	0	0	0
M15 240958	41530	11	6	16	12	9	17	NS	10	10	8	7	9	9	2	0	0	1	1	0	0	0	1
M15 240960	41531	11	6	16	15	7	16	NS	10	10	11	9	12	9	2	0	1	1	1	0	1	0	1
M15 240963	41533	10651	6	16	15	6	15	NS	10	10	8	8	8	6	2	0	0	1	1	0	0	0	1
M15 240988	41552	10651	6	16	11	7	16	NS	10	11	11	9	12		2	2	1	1	0	0	1	0	n/a
M16 240003	42475	11	6	16	12	10	18	NS	10	10	11	10	12	9	2	0	2	1	1	0	0	0	1
M16 240005	42476	10651	6	16	12	6	15	NS	9	9	11	9	11	12	2	0	0	0	0	0	1	0	1
M16 240011	42481	11	6	16	15	7	16	NS	10	10	10	9	11	11	2	0	1	1	1	0	1	0	0
M16 240013	42482	11	6	16	12	7	16	NS	10	11	10	9	12	10	2	0	1	1	0	0	1	0	0
M16 240014	42483	11	6	16	12	7	16	NS	7	9	8	8	9	10	2	0	0	1	0	0	0	0	0
M16 240016	42485	11	6	16	8	7	16	NS	11	10	10	10	12	14	2	2	1	0	1	0	0	0	0
M16 240017	42486	11	6	16	9	8	16	NS	10	10	11	9	11	12	2	0	1	1	1	0	1	0	1
M16 240020	42489	11	6	16	12	7	16	NS	9	10	11	9	10	11	2	0	1	0	1	0	1	1	0
M16 240031	42497	10651	6	16	12	7	16	NS	7	10	9	8	12	11	2	0	1	1	1	1	0	0	0

M16 240065	42525	10651	6	16	12			NS	10	11	11	10	12	10	2	0	n/a	1	0	0	0	0	0
M16 240070	42530	11	6	16	9	9	17	NS	12	11	11	9	11	8	2	0	2	0	0	0	1	0	0
M16 240116	42555	11	6	16	12	7	16	NS	9	11	10	9	12	10	2	0	0	0	0	0	1	0	0
M16 240123	42561	10651	6	16	16	12	20	NS	10	10	11	9	12	8	2	1	1	1	1	0	1	0	0
M16 240138	42573	10651	6	16	15	7	16	NS	9	10	8	9	13	10	2	0	1	0	1	0	1	1	0
M16 240154	42586	10651	6	16	12	7	16	NS	10	10	12	9	12	9	2	0	1	1	1	1	1	0	1
M16 240163	42595	11	6	16	9	7	16	NS	10	13	10	9	13	9	2	0	1	1	1	0	1	1	1
M16 240180	42605	11	6	16	12	7	16	NS	10	10	11	9	12	9	2	0	1	1	1	0	1	0	1
M16 240181	42606	11	6	16	6	6	15	NS	9	9	11	11	9		2	0	0	0	0	0	0	0	n/a
M16 240189	42612	11	6	16	9	7	16	NS	10	9	11	9	12	8	2	0	0	1	0	0	1	0	0
M16 240195	44684	11	6	16	12	7	16	NS	10	10	10	10	12	11	2	0	1	1	1	0	0	0	0
M16 240196	44685	11	6	16	10	7	16		10	10	11	9	10	11	2	1	1	1	1	0	1	1	0
M16 240198	44687	11	6	16		6	15	NS	9	9	11	9	11	9	2	n/a	0	0	0	0	1	0	1
M16 240231	44714	8621	6	16	9	7	16	NS	11	10	10	10	12	8	2	0	1	0	1	0	0	0	0
M16 240238	44718	11	6	16	12	6	15	NS	10	10	10	10	12	12	2	0	0	1	1	0	0	0	1
M16 240249	44727	10651	6	16	9	7	16	NS	9	11	11	9	13	10	2	0	1	0	0	0	1	1	0
M16 240271	44742	12208	6	16	12	7	16	NS	11	10	11	9	11	10	2	0	0	0	1	0	1	0	0
M16 240290	44753	11	6	16	15	12		NS	8	9	10	9	8	12	2	0	n/a	0	0	0	1	0	1
M16 240381	44768	11	6	16	12	9	17	NS	10	10	11	9	8	9	2	0	2	1	1	0	1	0	1
M16 240391	44777	11	6	16	11	8	17	NS	11	10	10	9	10	8	2	2	2	0	1	0	1	1	0
M16 240253	47016	11	6	16	9	7	16	NS	9	10	9	10	11	11	2	0	1	0	1	1	0	0	0
M16 240296	47019	11	6	16	11	8	17	NS	10	10	9	8	14	10	2	2	0	1	1	1	0	0	0
M16 240523	47271	11	6	16	8	8	16	NS	10	10	10	10	11	12	2	2	1	1	1	0	0	0	1
M16 240528	47274	10651	6	16	15			NS	10	10	11	9	13	9	2	0	n/a	1	1	0	1	1	1
M16 240529	47275	10651	6	16	15	10	18	NS	10	10	11	9	9	9	2	0	2	1	1	0	1	0	1
M16 240531	47277	10651	6	16	12	11	19	NS	10	10	11	9		9	2	0	1	1	1	0	1	n/a	1
M16 240535	47280	10215	6	16	12	10	19	NS	8	14	7	10	13	10	2	0	1	0	0	0	0	1	0
M16 240544	47289	11	6	16	12	7	16	NS	9	11	12	9	12	9	2	0	1	0	0	1	1	0	1
M16 240552	47294	11	6	16	9	10	18	NS	11	10	10	9	9	8	2	0	2	0	1	0	1	0	0
M16 240562	47296	11	6	16	12	7	16	NS	10	10	8	8	10	10	2	0	0	1	1	0	0	1	0

M16 240651	47322	11	6	16	10	7	16	NS	9	10	9	9	13	8	2	1	0	0	1	1	1	1	0
M16 240667	47329	11	6	16	9	7	16	NS	9	10	10	10	13	11	2	0	1	0	1	0	0	1	0
M16 240685	47338	11	6	16	11	7	16	NS	9	10	10	11	13	10	2	2	0	0	1	0	0	1	0
16.8704391	52862	11	6	16	9	7	16	NS	10	10	12	10	12	8	2	0	1	1	1	1	0	0	0
16.8709479	52880	11	12	19	12	8	17	NS	10	10	11	10	12	8	0	0	2	1	1	0	0	0	0
16.8709973	52885	11	6	16	12	7	16	NS	9	10	10	9	12	9	2	0	1	0	1	0	1	0	1
16.8710760	52889	11	6	16	12	10	18	NS	10	10	11	8	14	14	2	0	2	1	1	0	0	0	0
M16 240707	53053	10651	6	16	12	8	17	NS	10	7	7	10	14	6	2	0	2	1	1	0	0	0	1
M16 240721	53064	10651	6	16	9	7	16	NS	11	10	11	9	12	8	2	0	1	0	1	0	1	0	0
M16 240727	53067	11	6	16	12	6	15	NS	10	10	9	10	13	9	2	0	0	1	1	1	0	1	1
M16 240734	53072	11	6	16	12	7	16	NS	9	10	10	9	12	9	2	0	1	0	1	0	1	0	1
M16 240736	53074	10651	6	16	12	7	16	NS	11	10	10	9	9	8	2	0	1	0	1	0	1	0	0
M16 240738	53076	11	6	16	9	7	16	NS	9	10	13	9	13	12	2	0	1	0	1	0	1	1	1
M16 240756	53092	11	6	16	13	7	16	NS	10	10	15	10	12	11	2	1	1	1	1	1	0	0	0
M16 240757	53093	11	6	16	14	7	16	NS	10	10	12	10	12	9	2	2	1	1	1	1	0	0	1
M16 240794	53119	11	6	16	12	6	15	NS	10	10	9	13	9	9	2	0	0	1	1	1	0	0	1
M16 240800	53123	11	6	16	9	7	16	NS	10	10	12	10	13	8	2	0	1	1	1	1	0	1	0
M16 240843	53150	1287	6	16	9	7	16	NS	11	10	9	9	13	9	2	0	1	0	1	1	1	1	1
M16 240849	53154	11	5	15	9	9	17	NS	10	10	10	9	12	8	0	0	0	1	1	0	1	0	0
M16 240851	53156	11	6	16	8	6	16	NS	7	10	8	10	9	11	2	2	1	1	1	0	0	0	0
M16 240855	53159	13099	6	16	12	10	19	NS	9	10	9	9	9	10	2	0	1	0	1	1	1	0	0
M16 240865	53166	11	6	16	12	7	16	NS	10	10	12	10	15	8	2	0	1	1	1	1	0	0	0
M16 240867	53168	11	6	16	12	7	16	NS	10	10	11	10	12	13	2	0	1	1	1	0	0	0	0
M16 240869	53170	11	6	16	12	6	15	NS	9	9	12	9	12	10	2	0	0	0	0	1	1	0	0
M16 240873	53172	11	6	16	9	8	17	NS	10	10	8	10	9	6	2	0	2	1	1	0	0	0	1
M16 240879	53178	11	6	16	9	6	16	NS	8	8	7	8	10	10	2	0	1	0	0	0	0	1	0
M16 240897	53194	11	6	16	9	8	17	NS	10	10	13	10	13	12	2	0	2	1	1	0	0	1	1
M17 240006	53208	11	6	16	12	7	16	NS	10	11	9	8	9	11	2	0	1	1	0	1	0	0	0
M17 240013	53215	11	6	16	12	8	17	NS	8	9	9	9	7	11	2	0	2	0	0	1	1	1	0
M17 240020	53222	11	6	16	12	7	16	NS	9	7	10	10	9	10	2	0	1	0	1	0	0	0	0

M17 240021	53223	11	6	16	9	7	16	NS	10	11	12	11	12	13	2	0	1	1	0	1	0	0	0
M17 240022	53224	11	6	16	10	7	16	NS	11	10	12	10	15	12	2	1	1	0	1	1	0	0	1
M17 240029	53230	11	6	16	13	7	16	NS	9	10	10	9	13	12	2	1	1	0	1	0	1	1	1
M17 240030	53231	11	6	16	13	7	16	NS	10	11	11	10	12	9	2	1	1	1	0	0	0	0	1
M17 240036	53237	11	6	16	12	7	16	NS	10	10	14	10	12	10	2	0	1	1	1	0	0	0	0
M17 240071	53267	11	6	16	9	7	16	NS	10	10	11	9	12	10	2	0	1	1	1	0	1	0	0
M17 240073	53269	11	6	16	9	7	16	NS	9	10	9	10	14	10	2	0	1	0	1	1	0	0	0
M17 240084	53274	11	6	16	9	8	17	NS	10	8	9	8	11	11	2	0	2	1	0	1	0	0	0
M17 240100	53284	11	6	16	9	7	16	NS	10	10	9	10	13	8	2	0	1	1	1	1	0	1	0
M17 240120	53291	11	6	16	9	13	22	NS	7	10	7	10	9	10	2	0	0	1	1	0	0	0	0
M17 240127	53296	11	6	16	10	8	17	NS	10	11	10	9	13	9	2	1	2	1	0	0	1	1	1
M17 240135	53302	11	6	16	9	7	16	NS	10	10	12	11	13	12	2	0	1	1	1	1	0	1	1
M17 240138	53305	11	6	16	11	6	15	NS	10	10	11	10	12	13	2	2	0	1	1	0	0	0	0
M17 240140	53307	11	6	16	9	7	16	NS	9	9	11	9	11	11	2	0	1	0	0	0	1	0	0
M17 240162	53323	11	6	16	15	7	16	NS	10	10	12	10	12	16	2	0	1	1	1	1	0	0	0
M17 240167	53328	11	6	16	12	7	16	NS	10	10	12	10	12	12	2	0	1	1	1	1	0	0	1
M17 240180	53339	11	6	16	12	8	17	NS	10	10	10	9	14	8	2	0	2	1	1	0	1	0	0
M17 240196	53350	11	6	16	12	7	16	NS	10	11	11	9		10	2	0	1	1	0	0	1	n/a	0
M17 240204	53358	11	6	16	17	7	16	NS	8	9	9	10	9	11	2	0	1	0	0	1	0	0	0
M17 240218	53368	11	6	16	12	9	17	NS	10	10	9	10	12	8	2	0	0	1	1	1	0	0	0
M17 240227	53372	11	6	16	15	7	16	NS	10	13	9	11	12	8	2	0	1	1	1	1	0	0	0

Repeat Numbers for 2013 MenW cc11 Invasive Isolates Derived from Genome Sequence Data

		Type (ST)	(Poly C)	[illegible]	TAAA)	(Poly G)	[illegible]	(Poly C)	(Poly G)	(Poly C)	(Poly C)	(Poly G)	(PolyG)	(PolyG)	[illegible]	[illegible]	[illegible]	[illegible]	[illegible]	[illegible]	[illegible]	[illegible]	pilC2
M13 240015	28134	11	6	16	9	11	19	NS	9	10	10	10	12	11	2 0		1	0	1	0	0	0	0
M13 240246	28150	11	6	16	17	9	17	NS	9	10	11	9	11	10	2	0	0	0	1	0	1	0	0
M13 240269	28153	11	6	16	12	9	17	NS	8	10	9	9	9	11	2	0	0	0	1	1	1	0	0
M13 240283	28154	11	6	16	12	10	18	NS	10	10	11	9	12	10	2	0	2	1	1	0	1	0	0
M13 240436	28156	11	6	16	12	9	17	NS	9	9	11	9	11	9	2	0	0	0	0	0	1	0	1
M13 240446	28157	11	6	16	16	11	19	NS	11	10	11	9	13	12	2	1	1	0	1	0	1	1	1
M13 240491	29721	11	6	16	12	10	18	NS	9	10	11	9	12	11	2	0	2	0	1	0	1	0	0
M13 240545	29727	11	6	16		10	18	NS	10	10	11	9	12	10	2n/a		2	1	1	0	1	0	0
M13 240600	29736	11	6	16	13	9	17	NS	10	10	11	9	13	8	2	1	0	1	1	0	1	1	0
M13 240553	29737	11	6	16	21	9	17	NS	10	10	11	9	12	13	2	0	0	1	1	0	1	0	0
M13 240672	30145	11	6	16	9	9	17	NS	10	10	11	9	12	16	2	0	0	1	1	0	1	0	0
M13 240681	30148	11	6	16	12	10	18	NS	10	10	11	9	12	10	2	0	2	1	1	0	1	0	0
M13 240732	30153	11	6	16	16	9	17	NS	9	10	11	9	14	9	2	1	0	0	1	0	1	0	1
M14 240026	30160	11	6	16	15	10	18	NS	10	10	11	9	13	10	2	0	2	1	1	0	1	1	0
M14 240029	30161	11	6	16	15	10	18	NS	9	10	11	9	14	10	2	0	2	0	1	0	1	0	0
M14 240054	30167	11	6	16	9	10	18	NS	8	9	10	9	12	10	2	0	2	0	0	0	1	0	0
M14 240072	30168	11	6	16	15	10	18	NS	10	10	11	9	12	8	2	0	2	1	1	0	1	0	0
M14 240448	31169	11	6	16	15	11	19	NS	10	10	11	9	13	9	2	0	1	1	1	0	1	1	1
13.8552.X	35397	11	6	16	12	8	16	NS	9	10	11	9	13	12	2	0	0	0	1	0	1	1	1
M13 240531	35425	11	6	16	12	10	18	NS	8	9	8	9	13	8	2	0	2	0	0	0	1	1	0
M13 240705	35507	11	6	16	13	9	17	NS	8	10	8	9	11	13	2	1	0	0	1	0	1	0	0
M14 240126	35608	11	6	16	17	11	19	NS	10	10	11	9	12	8	2	0	1	1	1	0	1	0	0

M14 240142	35618	11	6	16	11	9	17	NS	10	10	11	10	13	13	2	2	0	1	1	0	0	1	0
M14 240178	35632	11	6	16	13	10	18	NS	9	10	10	8		11	2	1	2	0	1	0	0n/a		0
M14 240180	35634	11	6	16	14	10	18	NS	9	10	11	9		9	2	2	2	0	1	0	1n/a		1
M14 240204	35638	11	6	16	16	9	17	NS	10	10	12	9	11	11	2	1	0	1	1	1	1	0	0
M14 240257	35674	11	6	16	16	10	18	NS	11	10	11	9		11	2	1	2	0	1	0	1n/a		0
M14 240258	35675	11	6	16	12	9	17	NS	9	10	11	9	11	8	2	0	0	0	1	0	1	0	0
M14 240283	35690	11	6	16	10	11	19	NS	9	10	9	8	9	8	2	1	1	0	1	1	0	0	0
M14 240304	35702	11	6	16	12	11	19	NS	11		9	10	9	10	2	0	1	0n/a		1	0	0	0
M14 240351	35731	11	6	16	12	10	18	NS	10	10	11	9	12	8	2	0	2	1	1	0	1	0	0
M14 240389	35754	11	6	16	12	11	19	NS	10	10	10	9	12	8	2	0	1	1	1	0	1	0	0
M14 240468	35786	11	6	16	12	9	17	NS	9	10	10	11	11	10	2	0	0	0	1	0	0	0	0
M14 240478	35796	11	6	16	12	12	20	NS	10	8	10	10	9	10	2	0	1	1	0	0	0	0	0
M14 240485	35800	11	6	16	14			NS	9	10	8	9	11	11	2	2n/a		0	1	0	1	0	0
M14 240495	35809	11	6	16	12	9	17	NS	10	10	11	9		10	2	0	0	1	1	0	1n/a		0
M14 240496	35810	11	6	16		8	16	NS	8	9	8	9	8	10	2n/a		0	0	0	0	1	0	0
M14 240500	35811	11	6	16	12	9	17	NS	10	10	11	9	13	10	2	0	0	1	1	0	1	1	0
M14 240511	37681	11	6	16		9	17	NS	10	10	11	9	12	11	2n/a		0	1	1	0	1	0	0
M14 240517	37685	11	6	16	15	9	17	NS	10	10	11	9	15	10	2	0	0	1	1	0	1	0	0
M14 240524	37692	11	6	16	9	9	17	NS	10	10	11	9	12	9	2	0	0	1	1	0	1	0	1
M14 240532	37699	11	6	16	8	9	17	NS	8	10	9	9	9	10	2	2	0	0	1	1	1	0	0
M14 240550	37711	11	6	16	9	11	19	NS	9	10	9	9	11	10	2	0	1	0	1	1	1	0	0
M14 240555	37716	11	6	16	11	10	18	NS	10	10	11	9	11	11	2	2	2	1	1	0	1	0	0
M14 240556	37717	11	6	16	12	9	17	NS	10	10	11	9	12	9	2	0	0	1	1	0	1	0	1

M14 240562	37722	11	6	16	10	9	17	NS	10	10	11	10	11	8	2	1	0	1	1	0	0	0	0
M14 240573	37731	11	6	16	12	10	18	NS	10	10	11	9	14	10	2	0	2	1	1	0	1	0	0
M14 240581	37736	11	6	16	12	10	18	NS	10	11	11	9	13	10	2	0	2	1	0	0	1	1	0
M14 240587	37739	11	6	16	9	9	17	NS	9	11	9	9	9	12	2	0	0	0	0	1	1	0	1
M14 240602	37747	11	6	16	7	9	17	NS	9	10	11	8	12	9	2	1	0	0	1	0	0	0	1
M14 240604	37748	11	6	16	15	9	17	NS	10	10	11	9	12	11	2	0	0	1	1	0	1	0	0
M14 240618	37754	11	6	16	17	9	17	NS	9	10	11	9	12	9	2	0	0	0	1	0	1	0	1
M14 240621	37757	11	6	16	12	10	18	NS	9	10	11	9	13	9	2	0	2	0	1	0	1	1	1
M14 240624	37760	11	6	16	17	10	18	NS	10	10	11	9	11	12	2	0	2	1	1	0	1	0	1
M14 240628	37764	11	6	16	15	9	17	NS	8	10	8	7	9	10	2	0	0	0	1	0	0	0	0
M14 240633	37769	11	6	16		9	17	NS	10	10	11	9	11	13	2n/a		0	1	1	0	1	0	0
M14 240634	37770	11	6	16	12	9	17	NS	9	9	9	10	9	10	2	0	0	0	0	1	0	0	0
M14 240638	37774	11	6	16	12	10	18	NS	10	10	11	9	12	8	2	0	2	1	1	0	1	0	0
M15 240005	37793	11	6	16	12	11	19	NS	10	10	10	9	13	8	2	0	1	1	1	0	1	1	0
M15 240011	37798	11	6	16		8	16	NS	9	10	10	9	11	8	2n/a		0	0	1	0	1	0	0
M15 240012	37799	11	6	16	15	10	18	NS	9	11	11	9	17	10	2	0	2	0	0	0	1	0	0
M15 240042	37822	11	6	16	10	11	19	NS	10	10	11	9	11	10	2	1	1	1	1	0	1	0	0
M15 240055	37834	11	6	16	9	10	18	NS	10	10	10	9	13	12	2	0	2	1	1	0	1	1	1
M15 240080	37856	11	6	16	12	8	16	NS	9	10	8	9	11	8	2	0	0	0	1	0	1	0	0
M15 240085	37860	11	6	16	15	10	18	NS	10	10	11	10	12	10	2	0	2	1	1	0	0	0	0
M15 240089	37861	11	6	16	12	9	17	NS	10	10	11	9	12	11	2	0	0	1	1	0	1	0	0
M15 240091	37862	11	6	16	13	9	17	NS	10	10	11	9	12	16	2	1	0	1	1	0	1	0	0
M15 240097	37866	11	6	16		9	17	NS	9	10	10	9	11	10	2n/a		0	0	1	0	1	0	0

M15 240099	37868	11	6	16	15	10	18	NS	9	10	11	9	12	11	2	0	2	0	1	0	1	0	0
M15 240101	37870	11	6	16	12	10	18	NS	8	10	10	9	13	8	2	0	2	0	1	0	1	1	0
M15 240105	37873	11	6	16	12	10	18	NS	11	10	11	9	11	11	2	0	2	0	1	0	1	0	0
M15 240107	37875	11	6	16	12	10	18	NS	10	10	11	9	12	15	2	0	2	1	1	0	1	0	1
M15 240112	37879	11	6	16	10	9	17	NS	10	10	12	9	12	10	2	1	0	1	1	1	1	0	0
M15 240115	37882	11	6	16	12	10	18	NS	10	10	10	9	13	10	2	0	2	1	1	0	1	1	0
M15 240116	37883	11	6	16	12	9	17	NS	8	10	8	8	12	11	2	0	0	0	1	0	0	0	0
M15 240128	37891	11	6	16	12	10	18	NS	10	11	11	10	12	10	2	0	2	1	0	0	0	0	0
M15 240148	37907	11	6	16		9	17	NS	10	9	11	9	12	8	2n/a		0	1	0	0	1	0	0
M15 240193	37940	11	6	16	17	8	16	NS	10	10	11	9	12	11	2	0	0	1	1	0	1	0	0
M15 240195	37942	11	6	16	12	8	17	NS	10	10	10	9	12	8	2	0	0	1	1	0	1	0	0
M15 240207	37951	11	6	16	12	9	17	NS	10	10	11	9	13	8	2	0	0	1	1	0	1	1	0
M15 240216	37958	11	6	16	12	10	18	NS	10	10	10	9	12	11	2	0	2	1	1	0	1	0	0
M15 240221	37961	11	6	16		9	17	NS	9	10	11	9	11		2n/a		0	0	1	0	1	0	n/a
M15 240251	37983	11	6	16	9	9	18	NS	9	10	11	9	12	10	2	0	2	0	1	0	1	0	0
M15 240256	37986	11	6	16	9	9	17	NS	9	10	11	9	11	9	2	0	0	0	1	0	1	0	1
M15 240257	37987	11	6	16	11	10	18	NS	10	10	11	9	12	11	2	2	2	1	1	0	1	0	0
M15 240258	37988	11	6	16	12	9	17	NS	10	10	10	9	12	11	2	0	0	1	1	0	1	0	0
M15 240260	37990	11	6	16		10	18	NS	11	10	9	9	11	11	2n/a		2	0	1	1	1	0	0
M15 240263	37992	11	6	16	12	10	18	NS	9	10	10	10	11	8	2	0	2	0	1	0	0	0	0
M15 240287	38007	11	6	16	12	10	18	NS	10	10	11	9	12	10	2	0	2	1	1	0	1	0	0
M15 240288	38008	11	6	16	12	9	17	NS	8	10	9	9	9	9	2	0	0	0	1	1	1	0	1
M15 240291	38011	11	6	16	9	11	19	NS	9	11	11	9	12	10	2	0	1	0	0	0	1	0	0

M15 240300	38017	11	6	16	11	9	17	NS	11	10	11	9	14	11	2	2	0	0	1	0	1	0	0
M15 240301	38018	11	6	16	12	11	19	NS	10	10	9	11	13	10	2	0	1	1	1	1	0	1	0
M15 240316	38027	11	6	16	12	9	17	NS	9	10	11	9	12	8	2	0	0	0	1	0	1	0	0
M15 240329	38037	11	6	16	12	10	18	NS	9	9	11	9	12	12	2	0	2	0	0	0	1	0	1
M15 240332	38038	11	6	16	10	10	18	NS	9	10	11	9	11	8	2	1	2	0	1	0	1	0	0
M15 240382	38065	11	6	16	12			NS	9	10	10	9	12	8	2	0n/a		0	1	0	1	0	0
M15 240383	38066	11	6	16	13	9	17	NS	9	10	9	10	9	8	2	1	0	0	1	1	0	0	0
M15 240441	38079	11	6	16	11	10	18	NS	9	10	9	7	11	11	2	2	2	0	1	1	0	0	0
M15 240461	38085	11	6	16	11	10	18	NS	9	10	11	10	11	10	2	2	2	0	1	0	0	0	0
M15 240465	38088	11	6	16	15	9	18	NS	10	10	11	9	13	10	2	0	2	1	1	0	1	1	0
M15 240511	38101	11	6	16	12	10	18	NS	9	10	11	9	12	12	2	0	2	0	1	0	1	0	1
M15 240554	38106	11	6	16	12	10	18	NS	10	10	11	9	16	10	2	0	2	1	1	0	1	1	0
M15 240556	38108	11	6	16	10	9	17	NS	9	10	11	9	12	9	2	1	0	0	1	0	1	0	1
M15 240557	38109	11	6	16	12	9	17	NS	10	10	11	9	12	9	2	0	0	1	1	0	1	0	1
M15 240576	38115	11	6	16	10	9	17	NS	9	10	10	12	9	10	2	1	0	0	1	0	1	0	0
M15 240605	38151	5121	6	16		9	17	NS	9	9	7	9	9	11	2n/a		0	0	0	0	1	0	0
15.8705948	38306	11	6	16	9	10		NS	9	10	12	9	11	8	2	0n/a		0	1	1	1	0	0
15.8706012	38307	11	6	16	15	9	17	NS	9	10	12	9	11	10	2	0	0	0	1	1	1	0	0
15.8706090	38308	11	6	16	14	9	17	NS	9	9	12	10	9	10	2	2	0	0	0	1	0	0	0
15.8706113	38309	11	6	16	18	10	18	NS	9		11	8	10	9	2	0	2	0n/a		0	0	1	1
M15 240722	38520	11	6	16	9	9	17	NS		10	11	9			2	0	0n/a		1	0	1n/a		n/a
15.8701412	38997	11	6	16	9	9	17	NS	10	10	11	9	9	9	2	0	0	1	1	0	1	0	1
15.8703241	38998	11	6	16		9	17	NS	12	11	8	9	11	10	2n/a		0	0	0	0	1	0	0
15.8703378	38999	11	6	16	13	10	18	NS	10	8	8	11	12	13	2	1	2	1	0	0	0	0	0

M15 240629	39320	11	6	16	7	9	17	NS	9	10	10	9	11	10	2	1	0	0	1	0	1	0	0
M15 240643	39331	11	6	16	12	9	17	NS	10	9	10	9	11	11	2	0	0	1	0	0	1	0	0
M15 240648	39335	11	6	16	12	8	16	NS	9	10	9	10	9	10	2	0	0	0	1	1	0	0	0
M15 240651	39337	11	6	16	12	9	17	NS	10	10	11	9	12	15	2	0	0	1	1	0	1	0	1
M15 240658	39341	11	6	16	12	10	18	NS	9	10	12	9	13	8	2	0	2	0	1	1	1	1	0
M15 240659	39342	11	8	15	11	9	17	NS	10	10	11	8	11	11	0	2	0	1	1	0	0	0	0
M15 240661	39344	11	6	16	12	9	17	NS	10	12	11	9		10	2	0	0	1	0	0	1n/a		0
M15 240704	39352	11	6	16	12	9	17	NS	9	8	7	10	11	8	2	0	0	0	0	0	0	0	0
M15 240718	39356	11	6	16	12	9	17	NS	10	10	10	10	11	12	2	0	0	1	1	0	0	0	1
M15 240720	39358	11	6	16	17	10	18	NS	10	10	9	7	9	11	2	0	2	1	1	1	0	0	0
M15 240740	39368	11	6	16	12	10	18	NS	9	8	8	8	9	11	2	0	2	0	0	0	0	0	0
M15 240742	39369	11	6	16	12	9	17	NS	10	10	9	9	11	10	2	0	0	1	1	1	1	0	0
M15 240785	39401	11	6	16	14	9	17	NS	10	10	11	10	12	8	2	2	0	1	1	0	0	0	0
M15 240796	39410	11	6	16	12	9	17	NS	9	10	11	9	12	10	2	0	0	0	1	0	1	0	0
M15 240816	39421	11	6	16	12	12		NS	11	11	9	11	11	10	2	0n/a		0	0	1	0	0	0
M15 240818	39422	11	6	16	12	10	18	NS	9	10	11	9	12	12	2	0	2	0	1	0	1	0	1
M15 240819	39423	11	6	16	15	9	17	NS	10	10	11	9	12	10	2	0	0	1	1	0	1	0	0
M15 240829	39429	11	6	16	14	10		NS	11	10	11	9	12	15	2	2n/a		0	1	0	1	0	1
M15 240830	39430	11	6	16	10	9	17	NS	8	10	8	9	9	8	2	1	0	0	1	0	1	0	0
M15 240831	39431	11	6	16	9	10	18	NS	9	10	8	8	11	8	2	0	2	0	1	0	0	0	0
M15 240833	39433	11	6	16	12	9	17	NS	9	10	10	9	11	10	2	0	0	0	1	0	1	0	0
M15 240844	39435	11	6	16	12	8	16	NS	9	10	10	9	12	10	2	0	0	0	1	0	1	0	0
M15 240845	39436	11	6	16	18	9	17	NS	10	11	10	9	13	9	2	0	0	1	0	0	1	1	1

M15 240860	41458	11	6	16	8	9	17	NS	8	10	10	10	11	10	2	2	0	0	1	0	0	0	0
M15 240866	41463	11	6	16	14	9	17	NS	10	10	11	9	12	9	2	2	0	1	1	0	1	0	1
M15 240871	41467	11	6	16	15	8	16	NS	9	10	9	9	9	11	2	0	0	0	1	1	1	0	0
M15 240873	41469	11	6	16	11	9	17	NS	9	10	7	10	11	8	2	2	0	0	1	0	0	0	0
M15 240879	41473	11	6	16	12	8	16	NS	11	9	11	9		8	2	0	0	0	0	0	1n/a		0
M15 240888	41481	11	6	16	12			NS	8	10	9	9	10	9	2	0n/a		0	1	1	1	1	1
M15 240893	41483	11	6	16	18	10	18	NS	10	10	11	9	13	10	2	0	2	1	1	0	1	1	0
M15 240905	41494	11	6	16	11	9	17	NS	10	11	9	8	11	11	2	2	0	1	0	1	0	0	0
M15 240937	41516	11	6	16	11	10	18	NS	10	10	11	9	13	10	2	2	2	1	1	0	1	1	0
M15 240946	41522	11	6	16	8	11		NS	9	10	12	9	11	14	2	2n/a		0	1	1	1	0	0
M15 240952	41525	11	6	16	10	10	18	NS	10	10	10	9	12	10	2	1	2	1	1	0	1	0	0
M15 240978	41543	11	6	16	12	10	18	NS	8	10	7	9	9	9	2	0	2	0	1	0	1	0	1
M15 240981	41546	11	6	16	12	10	18	NS	9	10	10	8	14	8	2	0	2	0	1	0	0	0	0
M15 240983	41547	11	6	16	12	10	18	NS	9	9	10	9	9	15	2	0	2	0	0	0	1	0	1
M15 240985	41549	11	6	16	12	10	18	NS	9	10	10	9	12	15	2	0	2	0	1	0	1	0	1
M15 240995	41558	11	6	16	9	9	17	NS	10	10	8	9	11	8	2	0	0	1	1	0	1	0	0
M16 240008	42478	11	6	16	12	9	17	NS	10	10	9	9	13	12	2	0	0	1	1	1	1	1	1
M16 240010	42480	11	6	16	15	9	17	NS	11	10	11	9	10	8	2	0	0	0	1	0	1	1	0
M16 240015	42484	11	6	16	11	9	17	NS	9	10	10	9	12	11	2	2	0	0	1	0	1	0	0
M16 240024	42492	11	6	16	13	8	16	NS	9	10	11	9	13	11	2	1	0	0	1	0	1	1	0
M16 240027	42494	11	6	16	15	8	16	NS	7	11	8	11	12	16	2	0	0	1	0	0	0	0	0
M16 240052	42517	11	6	16	15	9	17	NS	10	10	10	9	11	10	2	0	0	1	1	0	1	0	0
M16 240055	42519	11	6	16	9	9	17	NS	9	11	10	9	9	10	2	0	0	0	0	0	1	0	0

M16 240066	42526	11	6	16	14	9	17	NS	10	10	11	9		12	2	2	0	1	1	0	1n/a		1
M16 240072	42532	11	6	16	12	9	17	NS	9	9	11	9	12	10	2	0	0	0	0	0	1	0	0
M16 240073	42533	11	6	16	12	11	19	NS	10	11	11	9	12	9	2	0	1	1	0	0	1	0	1
M16 240075	42535	11	6	16	12	9	17	NS	9	10	11	9	11	9	2	0	0	0	1	0	1	0	1
M16 240088	42545	11	6	16	12	8	16	NS	8	9	10	9	11	10	2	0	0	0	0	0	1	0	0
M16 240092	42549	11	11	18	13	11	19	NS	8	9	11	9	11	10	1	1	1	0	0	0	1	0	0
M16 240108	42551	11	6	16	12	9	17	NS	9	10	10	10	11	10	2	0	0	0	1	0	0	0	0
M16 240111	42552	11	6	16	15	8	16	NS	9	11	11	9	11	10	2	0	0	0	0	0	1	0	0
M16 240112	42553	11	6	16	9	11	19	NS	9	12	8	9	12	9	2	0	1	0	0	0	1	0	1
M16 240117	42556	11	6	16	8	10	18	NS	11	10	10	9	9	9	2	2	2	0	1	0	1	0	1
M16 240126	42564	11	6	16	12	10	18	NS	11	10	11	9	13	10	2	0	2	0	1	0	1	1	0
M16 240131	42568	11	6	16	12	10	18	NS	9	11	9	7	9	11	2	0	2	0	0	1	0	0	0
M16 240136	42572	11	6	16	12	10	18	NS	10	9	9	9	9	10	2	0	2	1	0	1	1	0	0
M16 240141	42576	11	6	16		11		NS	7	10	8	11	12	10	2n/a	n/a		1	1	0	0	0	0
M16 240152	42585	11	6	16	12	10	18	NS	9	10	11	9	14	10	2	0	2	0	1	0	1	0	0
M16 240226	44636	11	6	16	15	12	20	NS	9	9	9	8		11	2	0	1	0	0	1	0n/a		0
M16 240232	44638	11	8	15	8	9	17	NS	9	10	11	9	8	10	0	2	0	0	1	0	1	0	0
M16 240234	44640	11	6	16		10	18	NS	8	10	8	8	12	8	2n/a		2	0	1	0	0	0	0
M16 240197	44686	11	6	16	15	10	18	NS	9	10	8	9	9	8	2	0	2	0	1	0	1	0	0
M16 240214	44704	11	6	16	12	10	18	NS	10	10	10	9	11	10	2	0	2	1	1	0	1	0	0
M16 240219	44708	11	6	16	13	7	15	NS	10	9	10	10	12	9	2	1	0	1	0	0	0	0	1
M16 240239	44719	11	6	16	10	11	19	NS	10	10	11	10	12	11	2	1	1	1	1	0	0	0	0
M16 240242	44721	11	6	16	15	9	17	NS	11	10	11	9	13	9	2	0	0	0	1	0	1	1	1

M16 240246	44724	11	6	16	14	9		NS	11	10	11	9	12	11	2	2n/a		0	1	0	1	0	0
M16 240247	44725	11	6	16	12	10	18	NS	10	10	10	8	12	9	2	0	2	1	1	0	0	0	1
M16 240252	44730	11	6	16	10	10	18	NS	9	10	10	9	9	9	2	1	2	0	1	0	1	0	1
M16 240255	44733	11	6	16	9	10		NS	10	10	11	9	11	10	2	0n/a		1	1	0	1	0	0
M16 240260	44736	11	6	16	18	9	17	NS	8	9	7	10	12	9	2	0	0	0	0	0	0	0	1
M16 240267	44739	11	6	16	15	8	16	NS	9	10	10	10	10	11	2	0	0	0	1	0	0	1	0
M16 240268	44740	11	6	16	8	9	17	NS	9	10	11	9	9	11	2	2	0	0	1	0	1	0	0
M16 240361	44759	11	6	16	9	9	17	NS	9	12	9	7	13	9	2	0	0	0	0	1	0	1	1
M16 240365	44763	11	6	16	12	10	18	NS	8	10	10	9	11	11	2	0	2	0	1	0	1	0	0
M16 240368	44765	11	6	16	11	9	17	NS	10	10	11	9	12	9	2	2	0	1	1	0	1	0	1
M16 240370	44767	11	6	16	12	9	17	NS	10	8	9	11	12	8	2	0	0	1	0	1	0	0	0
M16 240382	44769	11	6	16	10	9	17	NS	10	10	10	9	12	10	2	1	0	1	1	0	1	0	0
M16 240386	44773	11	6	16	18	11		NS	9	10	9	8	11	9	2	0n/a		0	1	1	0	0	1
M16 240451	44820	11	6	16	12	9	17	NS	9	10	11	9	11	8	2	0	0	0	1	0	1	0	0
M16 240499	44828	11	6	16	12	10	18	NS	6	10	8	10	12	11	2	0	2	0	1	0	0	0	0
M16 240513	47262	11	6	16	10	11	19	NS	9	10	10	9	9	8	2	1	1	0	1	0	1	0	0
M16 240514	47263	11	6	16	11	12	20	NS	11	10	10	10	9	11	2	2	1	0	1	0	0	0	0
M16 240516	47265	11	5	15	10	9	17	NS	10	10	9	9	13	10	0	1	0	1	1	1	1	1	0
M16 240525	47273	11	6	16	10	8	16	NS	9	10	12	9	11	9	2	1	0	0	1	1	1	0	1
M16 240542	47287	12511	6	16	11	9	17	NS	10	10	8	8	11	10	2	2	0	1	1	0	0	0	0
M16 240565	47299	11	6	16	11	9	17	NS	8	10	10	9	14	10	2	2	0	0	1	0	1	0	0
M16 240567	47301	11	6	16	20	15	22	NS	10	10	11	9	11	11	2	0	0	1	1	0	1	0	0
M16 240570	47303	11	6	16	8	11	19	NS	10	10	11	9	11	12	2	2	1	1	1	0	1	0	1

M16 240573	47304	11	6	16	10	9	17	NS	10	11	12	10	9	10	2	1	0	1	0	1	0	0	0	0
M16 240626	47305	11	6	16	12	9	17	NS	9	10	10	10	12	11	2	0	0	0	1	0	0	0	0	
M16 240637	47311	11	6	16	12	10	18	NS	9	10	11	9	11	8	2	0	2	0	1	0	1	0	0	
M16 240656	47325	11	6	16	16	11	19	NS	9	8	10	9	13	8	2	1	1	0	0	0	1	1	0	
M16 240657	47326	11	6	16	15	10	18	NS	10	11	12	9	13	10	2	0	2	1	0	1	1	1	0	
M16 240672	47334	11	6	16	22	11	19	NS	9	9	9	9	12	10	2	0	1	0	0	1	1	0	0	
M16 240683	47336	11	6	16	9	9	17	NS	9	10	11	9	11	11	2	0	0	0	1	0	1	0	0	
M16 240684	47337	11	6	16	12	10		NS	10	9	11	8	12	8	2	0n/a		1	0	0	0	0	0	
M16 240700	47340	11	6	16	9	9	17	NS	10	11	11	9	12	10	2	0	0	1	0	0	1	0	0	
M16 240714	47344	11	6	16	15	10	18	NS	9	10	9	8	11	8	2	0	2	0	1	1	0	0	0	
14.8701242	51599	11	6	16	12	10	18	NS	11	11	9	10	12	11	2	0	2	0	0	1	0	0	0	
14.8703475	51608	11	6	16	11	10	18	NS	10	9	11	9	12	10	2	2	2	1	0	0	1	0	0	
14.8706990	52837	11	6	16	17	10	16	NS	10	10	11	9	12	11	2	0	0	1	1	0	1	0	0	
14.8707646	52838	11	6	16	13	8	16	NS	9	11	9	9	12	11	2	1	0	0	0	1	1	0	0	
15.8707666	52845	11	6	16	9	9	17	NS	9	10	11	9	12	11	2	0	0	0	1	0	1	0	0	
15.8708749	52846	11	6	16		9	17	NS	10	9	10	10	12	11	2n/a		0	1	0	0	0	0	0	
15.8708877	52847	11	6	16	14	11	19	NS	9	10	9	13	9	8	2	2	1	0	1	1	0	0	0	
15.8709301	52848	11	6	16	9	11	19	NS	11	10	11	9	14	10	2	0	1	0	1	0	1	0	0	
16.8700070	52850	11	6	16	11	10	18	NS	9	10	10	9	12	11	2	2	2	0	1	0	1	0	0	
16.8700175	52851	11	6	16		9	17	NS	11	10	11	9	9	11	2n/a		0	0	1	0	1	0	0	
16.8701581	52856	11	6	16	15	10	18	NS	10	10	11	9	11	9	2	0	2	1	1	0	1	0	1	
16.8701660	52857	11	6	16		9	17	NS	7	13	8	10	13	11	2n/a		0	1	1	0	0	1	0	
16.8701752	52858	11	6	16	17	9	17	NS	7	10	8	8	9	9	2	0	0	1	1	0	0	0	1	
16.8703048	52859	11	6	16	12	10		NS	10	12	11	9	13	10	2	0n/a		1	0	0	1	1	0	
16.8703541	52860	11	6	16	10	9	17	NS	9	10	11	9	12	10	2	1	0	0	1	0	1	0	0	
16.9703924	52861	11	6	16	12	9	17	NS	10	10	11	9	14	8	2	0	0	1	1	0	1	0	0	
16.8707104	52868	11	6	16	13	10	18	NS	9	11	10	8	14	11	2	1	2	0	0	0	0	0	0	

16.8707965	52872	11			10	8	16	NS	10	11	11	9	14	11	n/a		1	0	1	0	0	1	0	0
16.8708517	52874	11	6	16	12	9	17	NS	10	10	10	9	12	12	2	0	2	1	1	0	1	0	1
16.8710422	52886	11	6	16	7	10	18	NS	9	10	11	9	12	9	2	1	2	0	1	0	1	0	1
M16 240681	53050	11	6	16	7	9	17	NS	10	10	11	9	14	12	2	1	0	1	1	0	1	0	1
M16 240706	53052	11	6	16	18	9	17	NS	10	10	11	9	10	10	2	0	0	1	1	0	1	1	0
M16 240708	53054	11	11	18	12	10	18	NS	10	10	11	8	11	11	1	0	2	1	1	0	0	0	0
M16 240710	53056	11	6	16		11	19	NS	7	8	7	12	9	10	2n/a		1	1	0	0	1	0	0
M16 240712	53058	11	6	16	9	13	21	NS	7	10	6		15	8	2	0	0	1	1	0n/a		0	0
M16 240715	53060	11	6	16	9	10	18	NS	7	10	8	10	11	8	2	0	2	1	1	0	0	0	0
M16 240729	53069	11	6	16	19	9	17	NS	8	8	7	12	9	9	2	0	0	0	0	0	1	0	1
M16 240740	53078	11	6	16	12	10	18	NS	11	10	12	9	13	8	2	0	2	0	1	1	1	1	0
M16 240743	53080	11	6	16	6	10	18	NS	9	10	11	8	12		2	0	2	0	1	0	0	0	n/a
M16 240744	53081	11	6	16	9	10	18	NS	9	10	10	10	12	11	2	0	2	0	1	0	0	0	0
M16 240745	53082	11	6	16	9	9	17	NS	7	10	7	11	9	10	2	0	0	1	1	0	0	0	0
M16 240748	53084	11	9	16	11	9	17	NS	10	9	11	9	12	8	2	2	0	1	0	0	1	0	0
M16 240750	53086	11	6	16	12	8	16	NS	9	10	10	9	9	8	2	0	0	0	1	0	1	0	0
M16 240755	53091	11	6	16	17	9	17	NS	10	10	10	8	14	11	2	0	0	1	1	0	0	0	0
M16 240762	53096	11	6	16	12	9	17	NS	10	11	9	10	12	8	2	0	0	1	0	1	0	0	0
M16 240781	53110	11	6	16	12	10	18	NS	8	13	7	7	11	10	2	0	2	0	1	0	0	0	0
M16 240784	53113	11	6	16	12	11	17	NS	10	9	8	6	11	8	2	0	0	1	0	0	0	0	0
M16 240796	53121	11	7	17	15	9	17	NS	10	10	10	10	13	10	2	0	0	1	1	0	0	1	0
M16 240816	53133	11	6	16	14	11	19	NS	9	10	9	9	10	10	2	2	1	0	1	1	1	1	0
M16 240819	53135	11	6	16	9	10	17	NS	9	10	10	9	11	11	2	0	0	0	1	0	1	0	0

M16 240828	53139	11	6	16	9	9	17	NS	8	9	10	12	11	13	2	0	0	0	0	0	1	0	0
M16 240834	53141	11	6	16	9	11	19	NS	12	10	10	9	9	14	2	0	1	0	1	0	1	0	0
M16 240835	53142	11	6	16	12	9	17	NS	10	9	11	9	12	11	2	0	0	1	0	0	1	0	0
M16 240836	53143	11	6	16	12	9	17	NS	10	10	11	9	13	9	2	0	0	1	1	0	1	1	1
M16 240839	53146	11	6	16	15	9	17	NS	10	10	11	9	14	14	2	0	0	1	1	0	1	0	0
M16 240842	53149	11	5	15		9	17	NS	10	10	11	9	9	9	0n/a		0	1	1	0	1	0	1
M16 240860	53164	11	6	16	14	9	17	NS	11	10	11	10	13	9	2	2	0	0	1	0	0	1	1
M16 240862	53165	11	6	16	9	9	17	NS	11	9	8	9	12	8	2	0	0	0	0	0	1	0	0
M16 240866	53167	11	6	16	15	8	16	NS	8	7	9	10	9	11	2	0	0	0	1	1	0	0	0
M16 240877	53176	11	6	16	15	9	17		10	10	11	9	12	9	2	0	0	1	1	0	1	0	1
M16 240881	53179	11	6	16	12	9	17		8	10	9	7	9	8	2	0	0	0	1	1	0	0	0
M16 240885	53183	11	6	16	12	12	20		8	10	9	11	9	10	2	0	1	0	1	1	0	0	0
M16 240891	53188	11	6	16	12	9	17		9	10	9	8	11	8	2	0	0	0	1	1	0	0	0
M16 240893	53190	11	6	16	12	10	18		9	10	11	9	14	11	2	0	2	0	1	0	1	0	0
M16 240895	53192	11	6	16	17	9	17		10	10	11	9	12	10	2	0	0	1	1	0	1	0	0
M16 240900	53195	11	6	16	12	9	17		10	10	11	9	14	12	2	0	0	1	1	0	1	0	1
M16 240901	53196	11	6	16	12	11	19		8	10	9	9	11	10	2	0	1	0	1	1	1	0	0
M16 240904	53198	11	6	16	10	10	18		10	11	10	9	14	9	2	1	2	1	0	0	1	0	1
M16 240905	53199	11	6	16	9	10	18		8	10	11	8	9	7	2	0	2	0	1	0	0	0	0
M16 240906	53200	11	6	16	9	9	17		9	10	11	10	13	10	2	0	0	0	1	0	0	1	0
M17 240010	53212	11	6	16	15	9	17	NS	9	9	11	9	13	11	2	0	0	0	0	0	1	1	0
M17 240011	53213	11	6	16	12	11	19	NS	9	8	7	8	9	8	2	0	1	0	0	0	0	0	0
M17 240015	53217	11	6	16	9	9	17	NS	8	11	7	10	9	8	2	0	0	0	0	0	0	0	0

M17 240019	53221	11	6	16	12	10	18	NS	9	10	11	9	12	12	2	0	2	0	1	0	1	0	1
M17 240025	53226	11	6	16	12	10	18	NS	11	10	11	9	12	10	2	0	2	0	1	0	1	0	0
M17 240031	53232	11	6	16	9	10	18	NS	10	10	12	9	12		2	0	2	1	1	1	1	0	n/a
M17 240032	53233	11	6	16	17	10	18	NS	10	10	11	9	14	12	2	0	2	1	1	0	1	0	1
M17 240035	53236	11	6	16	12	11	19	NS	9	10	10	10	9	10	2	0	1	0	1	0	0	0	0
M17 240044	53244	11	6	16	12	10	18	NS	10	10	11	9	12	11	2	0	2	1	1	0	1	0	0
M17 240049	53249	11	6	16	10	11		NS	9	10	10	9	12	12	2	1	n/a	0	1	0	1	0	1
M17 240052	53252	11	6	16	9	9	17	NS	10	10	11	9	12	9	2	0	0	1	1	0	1	0	1
M17 240057	53255	11	6	16	16	9	17	NS	9	10	11	9	14	8	2	1	0	0	1	0	1	0	0
M17 240058	53256	11	6	16	17	9	17	NS	9	10	10	10	11	10	2	0	0	0	1	0	0	0	0
M17 240063	53259	11	6	16		8	16	NS	10	10	11	9	14	9	2	n/a	0	1	1	0	1	0	1
M17 240068	53264	11	6	16	9	9	17	NS	10	10	11	9		12	2	0	0	1	1	0	1	n/a	1
M17 240076	53270	11	6	16	12	9	17	NS	10	12	8	9	9	10	2	0	0	1	0	0	1	0	0
M17 240085	53275	11	6	16	12	9	17	NS	8	11	7	9	9	6	2	0	0	0	0	0	1	0	1
M17 240092	53279	11	6	16	12	9	17	NS	8	11	10	9	9	10	2	0	0	0	0	0	1	0	0
M17 240103	53287	11	6	16	9	10	18	NS	7	12	9	11	11	10	2	0	2	1	0	1	0	0	0
M17 240122	53292	11	6	16	12	9	17	NS	9	10	10	9	12	10	2	0	0	0	1	0	1	0	0
M17 240129	53297	11	6	16	15	8	16	NS	10	10	10	10	11	8	2	0	0	1	1	0	0	0	0
M17 240133	53300	11	6	16	10	12	20	NS	10	10	11	9	12	10	2	1	1	1	1	0	1	0	0
M17 240134	53301	11	6	16	13	10	18	NS	11	10	11	9	14	11	2	1	2	0	1	0	1	0	0
M17 240141	53308	11	6	16	12	10	18	NS	9	11	8	10	8	8	2	0	2	0	0	0	0	0	0
M17 240142	53309	11	6	16	12	10	18	NS	9	9	9	8	12	10	2	0	2	0	0	1	0	0	0
M17 240149	53315	11	6	16	15	7	15	NS	8	8	10	12	9	8	2	0	0	0	0	0	1	0	0

M17 240150	53316	11	6	16	8	10	18	NS	10	10	11	9	14		2	2	2	1	1	0	1	0	n/a
M17 240161	53322	11	6	16	12	9	17	NS	14	11	11	9	12	9	2	0	0	0	0	0	1	0	1
M17 240164	53325	11	6	16	20	9	17	NS	8	10	9	13	11	10	2	0	0	0	1	1	0	0	0
M17 240179	53338	11	6	16	12	8	16	NS	10	10	11	9	13	13	2	0	0	1	1	0	1	1	0
M17 240181	53340	11	6	16	9	11	19	NS	10	10	11	9	15	8	2	0	1	1	1	0	1	0	0
M17 240182	53341	11	6	16	13	10	18	NS	10	10	11	10	14	10	2	1	2	1	1	0	0	0	0
M17 240184	53343	11	6	16	12	10	18	NS	9	14	9	9	9	10	2	0	2	0	0	1	1	0	0
M17 240185	53344	11	6	16	12	11	19	NS	9	10	10	9	12	11	2	0	1	0	1	0	1	0	0
M17 240192	53346	13097	10	17	12			NS	9	9	8	8	13	8	2	0n/a		0	0	0	0	1	0
M17 240193	53347	11	6	16	12	9	17	NS	11	10	12	9	12	10	2	0	0	0	1	1	1	0	0
M17 240198	53352	11	6	16	12	7	15	NS	9	10	9	9	11	9	2	0	0	0	1	1	1	0	1
M17 240200	53354	11	6	16	16	11	19	NS	11	10	9	9	14	10	2	1	1	0	1	1	1	0	0
M17 240201	53355	11	6	16	9	9	17	NS	10	10	11	9	14	11	2	0	0	1	1	0	1	0	0
M17 240207	53360	11	6	16	15	9	17	NS	9	10	10	9	16	8	2	0	0	0	1	0	1	1	0
M17 240214	53365	11	6	16	17	9	17	NS	8	10	9	9	9	8	2	0	0	0	1	1	1	0	0
M17 240217	53367	11	6	16	12	9	17	NS	9	10	10	13	9	10	2	0	0	0	1	0	0	0	0
M17 240221	53369	11	6	16	12	10	18	NS	9	9	8	8	13	9	2	0	2	0	0	0	0	1	1
M17 240226	53371	11	6	16	15	9	17	NS	10	10	11	9	13	11	2	0	0	1	1	0	1	1	0

Source Data for Invasive Meningococcal Isolates

Source	Strains
Blood	M10 240514, M10 240671, M10 240817, M10 240821, M11 240035, M11 240057, M11 240067, M11 240099, M11 240168, M11 240305, M11 240403, M11 240427, M12 240156, M12 240160, M11 240486, M11 240726, M11 240798, M11 240802, M11 240953, M11 240975, M12 240004, M12 240016, M12 240021, M12 240027, M12 240067, M12 240095, M12 240125, M12 240127, M12 240133, M12 240144, M12 240240, M12 240317, M12 240324, M12 240337, M12 240640, M12 240657, M12 240663, M12 240702, M12 240751, M12 240754, M12 240774, M12 240826, M12 240895, M12 240898, M13 240015, M13 240025, M13 240028, M13 240056, M13 240066, M13 240077, M13 240109, M13 240114, M13 240158, M13 240168, M13 240185, M13 240223, M13 240225, M13 240246, M13 240247, M13 240251, M13 240269, M13 240283, M13 240436, M13 240446, M13 240457, M13 240464, M13 240467, M13 240469, M13 240473, M13 240482, M13 240491, M13 240555, M13 240591, M13 240603, M13 240604, M13 240545, M13 240558, M13 240594, M13 240583, M13 240510, M13 240633, M13 240634, M13 240538, M13 240515, M13 240600, M13 240553, M13 240635, M13 240637, M13 240649, M13 240655, M13 240657, M13 240664, M13 240667, M13 240668, M13 240672, M13 240678, M13 240680, M13 240681, M13 240724, M13 240726, M13 240730, M13 240732, M14 240001, M14 240002, M14 240007, M14 240013, M14 240019, M14 240022, M14 240026, M14 240029, M14 240031, M14 240042, M14 240043, M14 240052, M14 240053, M14 240054, M14 240072, M14 240447, M14 240448, M13 240531, M13 240705, M14 240090, M14 240116, M14 240126, M14 240135, M14 240145, M14 240147, M14 240150, M14 240178, M14 240180, M14 240199, M14 240204, M14 240211, M14 240223, M14 240245, M14 240253, M14 240257, M14 240258, M14 240278, M14 240279, M14 240282, M14 240283, M14 240299, M14 240304, M14 240337, M14 240343, M14 240346, M14 240351, M14 240389, M14 240391, M14 240406, M14 240463, M14 240468, M14 240470, M14 240474, M14 240478, M14 240485, M14 240486, M14 240492, M14 240494, M14 240495, M14 240496, M14 240500, M14 240394, M14 240502, M14 240504, M14 240511, M14 240517, M14 240522, M14 240524, M14 240525, M14 240532, M14 240534, M14 240545, M14 240548, M14 240550, M14 240555, M14 240556, M14 240562, M14 240572, M14 240573, M14 240574, M14 240581, M14 240585, M14 240587, M14 240593, M14 240601, M14 240602, M14 240604, M14 240605, M14 240618, M14 240621, M14 240624, M14 240628, M14 240633, M14 240634, M14 240638, M14 240649, M15 240005, M15 240010, M15 240011, M15 240012, M15 240015, M15 240021, M15 240022, M15 240027, M15 240029, M15 240041, M15 240042, M15 240048, M15 240055, M15 240066, M15 240078, M15 240080, M15 240081, M15 240085, M15 240089, M15 240091, M15 240097, M15 240099, M15 240101, M15 240105, M15 240107, M15 240112, M15 240114, M15 240115, M15 240116, M15 240127, M15 240128, M15 240130, M15 240141, M15 240144, M15 240148, M15 240151, M15 240158, M15 240172, M15 240181, M15 240185, M15 240187, M15 240195, M15 240207, M15 240216, M15 240221, M15 240242, M15 240244, M15 240248, M15 240256, M15 240257, M15 240258, M15 240260, M15 240263, M15 240277, M15 240278, M15 240280, M15 240282, M15 240284, M15 240287, M15 240288, M15 240300, M15 240301, M15 240310, M15 240316, M15 240326, M15 240329, M15 240332, M15 240376, M15 240380, M15 240381, M15 240382, M15 240383, M15 240384, M15 240438, M15 240441, M15 240461, M15 240465, M15 240477, M15 240554, M15 240556, M15 240576, M15 240605, M15 240722, M15 240629, M15 240638, M15 240643, M15 240648, M15 240651, M15 240657, M15 240658, M15 240659, M15 240661, M15 240662, M15 240664, M15 240718, M15 240720, M15 240730, M15 240740, M15 240742, M15 240746, M15 240762, M15 240778,

	M15 240779, M15 240782, M15 240788, M15 240794, M15 240796, M15 240797, M15 240798, M15 240816, M15 240819, M15 240829, M15 240830, M15 240831, M15 240833, M15 240844, M15 240848, M15 240852, M15 240753, M15 240856, M15 240860, M15 240864, M15 240866, M15 240871, M15 240873, M15 240876, M15 240879, M15 240885, M15 240888, M15 240896, M15 240918, M15 240936, M15 240937, M15 240946, M15 240952, M15 240958, M15 240963, M15 240978, M15 240983, M15 240985, M15 240988, M15 240995, M16 240003, M16 240005, M16 240008, M16 240010, M16 240011, M16 240013, M16 240014, M16 240015, M16 240016, M16 240017, M16 240020, M16 240024, M16 240027, M16 240031, M16 240052, M16 240065, M16 240070, M16 240072, M16 240073, M16 240075, M16 240088, M16 240092, M16 240108, M16 240111, M16 240112, M16 240116, M16 240117, M16 240123, M16 240126, M16 240131, M16 240136, M16 240138, M16 240141, M16 240154, M16 240163, M16 240180, M16 240181, M16 240189, M16 240226, M16 240232, M16 240234, M16 240195, M16 240196, M16 240197, M16 240198, M16 240214, M16 240219, M16 240231, M16 240238, M16 240239, M16 240242, M16 240246, M16 240247, M16 240252, M16 240255, M16 240260, M16 240267, M16 240268, M16 240271, M16 240361, M16 240365, M16 240370, M16 240381, M16 240382, M16 240386, M16 240391, M16 240451, M16 240499, M16 240253, M16 240296, M16 240513, M16 240514, M16 240516, M16 240523, M16 240525, M16 240528, M16 240529, M16 240531, M16 240535, M16 240542, M16 240544, M16 240552, M16 240562, M16 240565, M16 240567, M16 240570, M16 240573, M16 240626, M16 240637, M16 240651, M16 240656, M16 240657, M16 240667, M16 240672, M16 240683, M16 240684, M16 240685, M16 240700, M16 240681, M16 240706, M16 240707, M16 240708, M16 240710, M16 240712, M16 240715, M16 240721, M16 240729, M16 240734, M16 240736, M16 240738, M16 240740, M16 240743, M16 240744, M16 240745, M16 240748, M16 240755, M16 240756, M16 240757, M16 240762, M16 240781, M16 240784, M16 240794, M16 240796, M16 240800, M16 240816, M16 240819, M16 240828, M16 240834, M16 240835, M16 240839, M16 240842, M16 240843, M16 240849, M16 240851, M16 240855, M16 240862, M16 240865, M16 240866, M16 240867, M16 240869, M16 240873, M16 240877, M16 240879, M16 240881, M16 240885, M16 240891, M16 240893, M16 240895, M16 240897, M16 240900, M16 240901, M16 240904, M16 240905, M16 240906,
CSF	M11 240417, M14 240082, M14 240142, M15 240271, M15 240785, M15 240845, M15 240905, M15 240960, M15 240981, M16 240727
Other	M11 240389, M13 240238, M14 240482, M15 240189, M15 240193, M15 240251, M15 240334, M15 240375, M15 240511, M15 240557, M15 240640, M15 240704, M15 240818, M15 240826, M15 240919, M15 240940, M16 240055, M16 240152, M16 240249, M16 240290, M16 240368, M16 240750, M16 240836, M16 240860, M15 240291, M16 240066, M16 240714

List of Invasive MenW Isolates for East Midlands and Random Selection

Category	Isolates (names or BIGSDB ID number
East Midlands	M10 240514, M11 240099, M12 240663, M13 240015, M13 240185, M13 240633, M13 240600, M13 240553, M13 240635, M13 240726, M14 240019, M14 240043, M14 240257, M14 240299, M14 240525, M14 240534, M14 240573, M14 240605, M15 240027, M15 240029, M15 240181, M15 240193, M15 240251, M15 240271, M15 240280, M15 240329, M15 240794, M15 240844, M15

	240918, M15 240988, M15 240995, M16 240066, M16 240070, M16 240239, M16 240290, M16 240296, M16 240528, M16 240667, M16 240683, M16 240743, M16 240796, M16 240800, M17 240030, M17 240031, M17 240071, M17 240140, M17 240185
Random Selection	28125, 53231, 38034, 28136, 30162, 37881, 35670, 39337, 29730, 42561, 53287, 47329, 31168, 28142, 52857, 44721, 42555, 35622, 28128, 53092, 51599, 47305, 37951, 38076, 37701, 39326, 20196, 37990, 38088, 35675, 53080, 30141, 28159, 35800, 47304, 39365, 28137, 35722, 20057, 53270, 39328, 35686, 37739, 44724, 20216, 53315, 53339, 37822, 44773, 42494, 53350, 28164, 35618, 28158
