# Supplementary material for: Potentiation of Phase Variation in Multiple Outer-Membrane Proteins During Spread of the Hyperinvasive Neisseria meningitidis Serogroup W ST-11 Lineage
Source: J Infect Dis. 2019 May 23;220(7):1109–17. doi: 10.1093/infdis/jiz275 (PMC6735796; doi:10.1093/infdis/jiz275)
Supplement: jiz275_suppl_Supplementary_Data_Figure_1 [file jiz275_suppl_supplementary_data_figure_1.docx]

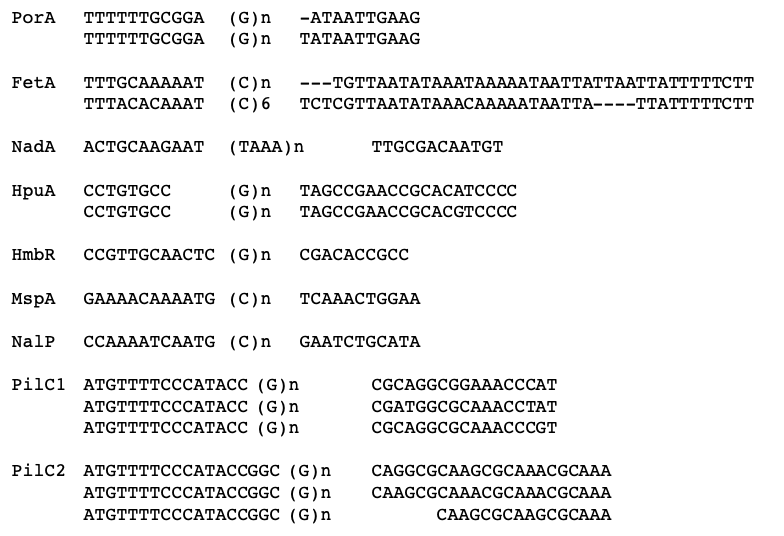


Supplementary Figure 1. Alignment of sequences flanking repetitive tracts. The sequences flanking the repetitive tracts were examined in multiple MenW ST-11 UK isolates for the genes encoding seven single-copy OMPs, PilC1 and PilC2. The sequences on either side of the repeats (i.e. polyC, polyG or 5’TAAA) are shown as changes in these regions can alter the repeat number associated with the ON or OFF expression states or the activity of a promoter element. Both sequences are shown where variation in these sequences was detected. Note that the repetitive tracts for PorA, FetA and NadA are located within the promoter regions while the other tracts are present in the reading frame.
