# Supplementary material for: Potentiation of Phase Variation in Multiple Outer-Membrane Proteins During Spread of the Hyperinvasive Neisseria meningitidis Serogroup W ST-11 Lineage
Source: J Infect Dis. 2019 May 23;220(7):1109–17. doi: 10.1093/infdis/jiz275 (PMC6735796; doi:10.1093/infdis/jiz275)
Supplement: jiz275_suppl_Supplementary_Data_Figure_2 [file jiz275_suppl_supplementary_data_figure_2.docx]

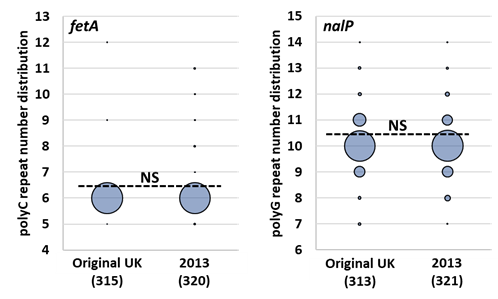


Supplementary Figure 2. Repeat number distributions for *feta* and *nalP*. Repeat numbers were determined by bioinformatics analyses of whole genome sequence data (*fetA*, *nalP*) for multiple disease isolates of the original-UK and 2013- strains of the MenW ST-11 South American strain lineage. ‘Bubbles’ represent the proportion of isolates (see brackets for the total number of isolates) with a particular repeat number. Dotted line, modal repeat number; a Fisher’s exact test was performed on the numbers of isolates with repeat numbers above or below, respectively, the mode for the original-UK versus 2013-strain. NS, non-significant.
