# Supplementary material for: Potentiation of Phase Variation in Multiple Outer-Membrane Proteins During Spread of the Hyperinvasive Neisseria meningitidis Serogroup W ST-11 Lineage
Source: J Infect Dis. 2019 May 23;220(7):1109–17. doi: 10.1093/infdis/jiz275 (PMC6735796; doi:10.1093/infdis/jiz275)
Supplement: jiz275_suppl_Supplementary_Data_Figure_3 [file jiz275_suppl_supplementary_data_figure_3.docx]

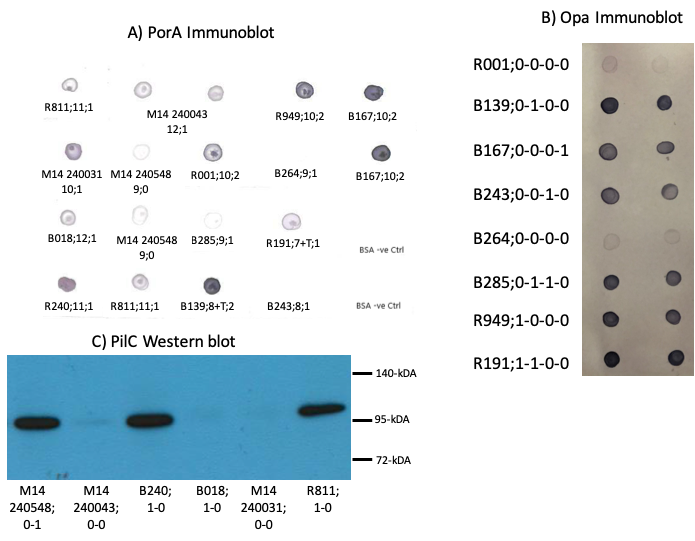


Supplementary Figure 3. PorA, Opa and PilC immunoblots. Meningococcal strains were grown overnight on BHI agar plates. For the immunoblots, bacterial suspensions were incubated at 56^o^C for 30 minutes and then aliquots were applied to a nitrocellulose filter. For the Western blot, bacterial pellets were resuspended in 1X SDS loading buffer, heated at 65^o^C for 15 minutes, and subject to electrophoresis on a 10% polyacrylamide gel prior to overnight transfer at 4^o^C to a PVDF membrane. A) PorA immunoblot was probed with a 1:1000 dilution of a P1.5 PorA mouse monoclonal antibody followed by a 1:2000 dilution of goat anti-mouse alkaline phosphatase-conjugated antibody (Original isolates M14 240031; post-2013 isolates, . B) Opa immunoblot was probed with a 1:1000 dilution of a rabbit polyclonal antibody raised against a purified partially-denatured Opa protein followed by a 1:2000 dilution of goat anti-rabbit alkaline phosphatase-conjugated antibody. C) PilC western blot was probed with a 1:500 dilution of 4B5_10, a PilC-specific mouse monoclonal antibody, followed by a 1:6000 dilution of goat anti-mouse horse radish peroxide-conjugated antibody. Each dot or lane is identified by the strain plus additional information:- A) PorA repeat number with (+T) or without an additional T immediately downstream of the repeat tract (see Supplementary Figure 1) followed by the expected expression state (i.e. 0, low; 1, intermediate; 2, high); B) Opa, phasotype for the OpaA-OpaB-OpaD-OpaJ proteins (1=ON, 0=OFF, so that 0-1-0-0 designates a strain expressing only the OpaB protein); 3) PilC, phasotype for the PilC1-PilC2 proteins (1=ON, 0=OFF, so that 0-1 designates a strain expressing only the PilC2 protein). Note that the Opa and PilC antisera cross-react with all variants of these proteins.
