# Supplementary material for: Potentiation of Phase Variation in Multiple Outer-Membrane Proteins During Spread of the Hyperinvasive Neisseria meningitidis Serogroup W ST-11 Lineage
Source: J Infect Dis. 2019 May 23;220(7):1109–17. doi: 10.1093/infdis/jiz275 (PMC6735796; doi:10.1093/infdis/jiz275)
Supplement: jiz275_suppl_Supplementary_Data_Figure_4 [file jiz275_suppl_supplementary_data_figure_4.docx]

Supplementary Figure 4. Comparison of expression states for multiple PV genes between MenW ST-11 carriage isolates and subsets of invasive isolates. Expression states for each phase-variable gene were predicted from repeat numbers (see text); transcriptional SSRs (*porA*, *fetA*, and *nadA*) were coded as low (0), intermediate (1) and high (2) expression; translational SSRs were coded as ON (1) or OFF (0). Phasotypes were derived from these expression states for the seven scOMPs and the two PilC. For the scOMPs, phasotypic scores were derived by simple addition of the expression states of each phasotype. Significance values were obtained using the numbers of isolates with either a particular expression state or phasotypes/phasotypic scores above and below the dotted line. The panels on the left show a comparison of all the invasive isolates from both strains to the isolates obtained in the 2015-2016 carriage study (predominantly the 2013-strain. The middle panels show a comparison of invasive isolates obtained in 2015-2016 from the East Midlands to the carriage study isolates (note that this study was conducted at the University of Nottingham which is in the East Midlands area). The panels of right show a comparison between 45 randomly-selected 2013-strain isolates to the carriage isolates. The significance values for the PilC comparisons were identical across all of these comparisons indicating that the differences were not due solely to the large number of invasive isolates or to the regional localisation of the carriage isolates.
