# Supplementary material for: Potentiation of Phase Variation in Multiple Outer-Membrane Proteins During Spread of the Hyperinvasive Neisseria meningitidis Serogroup W ST-11 Lineage
Source: J Infect Dis. 2019 May 23;220(7):1109–17. doi: 10.1093/infdis/jiz275 (PMC6735796; doi:10.1093/infdis/jiz275)
Supplement: jiz275_suppl_Supplementary_Data_Tables [file jiz275_suppl_supplementary_data_tables.docx]

Supplementary Table 1. Oligonucleotide primers for the GeneScan analyses of the *pilC* and *opa* genes of MenW ST-11 clonal complex isolates

| Gene | Primer Name | Primer Sequence |
| --- | --- | --- |
| *pilC1* | pilC1-for | TTTCCGCCATACCGCGCTTTATG |
|  | pilC1-rev-tract | GTACCGCTTTGTCGGGAAACA |
| *pilC2* | pilC2-for | TCCGCCATACCGCGCTTTATA |
|  | pilC2-rev-tract | AAGGGCATTTTTGCGCTCGG |
| *Opa* (all) | ND009_Opa_F | TTGAAACATCGCCCCAAACC |
| *opaA* | ND012_Opa_Loci3_R | CCCTGTTGCCATTGCTATGTC |
| *opaB/opaD* | ND011_Opa_Loci2_R | TGGGGGTCGGACCTTGTAGG |
| *opaJ* | ND010_Opa_Loci1_R | GGTCGAATCGATGCTGTGTCTG |

Supplementary Table 2. Alleles for scOMP in MenW ST-11 carriage and disease isolate

|  | **Isolate** | |
| --- | --- | --- |
| **scOMP Locus** | **Carriage (101)^1^** | **Invasive (636)^1^** |
| NEIS1963 (*fetA*) | 13 (86), 1468 (14), ND^2^ | 13 (570), 488 (19), 1254 (7), 1924 (2), 0 (2), 20 (2), 309 (2), 1240 (2), 1519 (2), 1520 (2), 1748 (2), 1915 (2), 151, 756, 1105, 1153, 1709, 1710, 1910, 2239, 2242, 2409, ND^2^ (11) |
| *hmbR* | 1 (98), ND^2^ (3) | 1 (595), ND^2^ (41) |
| NEIS1943 (*hpuA*) | 167 (28), 110 (4), 175, ND^2^ (68) | 167 (154), 175 (17), 165 (5), 110 (2), 618, ND^2^ (456) |
| NEIS1974 (*mspA*) | 1 (101) | 1 (632), 127, 128, ND^1^ (2) |
| NEIS1969 (*nadA*) | 5 (99), 0, ND^2^ | 5 (63), 3, 146, 207, 245, ND^2^ (2) |
| NEIS1943 (*nalP*) | 2 (53), 165 (7), ND^2^ (41) | 2 (441), 229 (7), 185 (3), 137, 342, 346, 347, 746, 747, ND^2^ (179) |
| NEIS1364 (*porA*) | 1 (95), 384 (2), 1482, ND^2^ (3) | 1 (590), 603 (5), 393 (3), 0 (2), 59 (2), 142 (2), 1558 (2), 1692 (2), 4, 12, 322, 387, 930, 941, 980, 1040, 1215, 1327, 1331, 1337, 1338, 1339, 1340, 1520, 1556, 1560, 1603, ND^2^ (9) |

^1^Numbers in brackets indicate the total number of isolates; ^2^ND, no data

Supplementary Table 3. Summary of comparison of repeat numbers derived by GeneScan versus whole genome sequence analysis

| Gene | Total Isolates Tested | | Identical Repeat Numbers | | Repeat Numbers in Non-Matching Isolates^1^ | |
| --- | --- | --- | --- | --- | --- | --- |
|  | Disease | Carriage | Disease | Carriage | Disease | Carriage |
| *nadA* | 88 | 37 | 77 (88%) | 33 (89%) | 10(2),13,14,15,17(5),21 | 15(4) |
| *hmbR* | 92 | 42 | 85 (92%) | 41 (98%) | 9(3),10(2),11(2) | 12 |
| *fetA* | 91 | 44 | 91 (100%) | 43 (98%) | - | 12 |
| *hpuA* | 90 | 39 | 88 (98%) | 39 (100%) | 10,11 | None |
| *mspA* | ND^2^ | 43 | - | 40 (93%) | - | 8,9,11 |
| *nalP* | ND | 45 | - | 45 (100%) | - | None |
| *pilC1* | 77 | 40 | 59 (77%) | 36 (90%) | 9,10,11(6),12,13(4),15(3),20(2) | 10,13(3) |
| *pilC2* | 79 | 38 | 68 (86%) | 35 (92%) | 8(2),9(2),10,11,12(3),13,16 | 9,11,13 |

^1^Brackets indicate the number of isolates mis-matched for this repeat number; ^2^ND, no data.

Supplementary Table 4. Comparison of repeat numbers for UK invasive versus carriage isolates of the MenW ST-11 lineage for both the original and 2013- strains.

| Gene | Modal repeat number | Repeat number | Original-strain | | | 2013-strain | | |
| --- | --- | --- | --- | --- | --- | --- | --- | --- |
|  |  |  | Invasive^1^ | Carriage^1^ | P value^2^ | Invasive^1^ | Carriage^1^ | P value^2^ |
| *porA* | 7 | >7 | 93 (30%) | 15 (27%) | 0.75 | 314 (99%) | 46 (100%) | 1.0 |
|  |  | ≤7 | 216 | 40 |  | 3 | 0 |  |
| *nadA* | 12 | >12 | 50 (16%) | 11 (23%) | 0.303 | 89 (29%) | 24 (53%) | 0.002 |
|  |  | ≤12 | 256 | 37 |  | 213 | 21 |  |
| *fetA* | 6 | >6 | 2 (0.6%) | 0 (0%) | 1.0 | 7 (2%) | 15 (33%) | <0.0001 |
|  |  | ≤6 | 313 | 55 |  | 313 | 31 |  |
| *hpuA* | 10 | >10 | 208 (66%) | 36 (65%) | 0.878 | 157 (49%) | 23 (50%) | 1.0 |
|  |  | ≤10 | 105 | 19 |  | 163 | 23 |  |
| *hmbR* | 9 | >9 | 101 (32%) | 13 (23%) | 0.268 | 61 (19%) | 13 (28%) | 0.169 |
|  |  | ≤9 | 213 | 42 |  | 259 | 33 |  |
| *nalP* | 10 | >10 | 51 (16%) | 18 (32%) | 0.008 | 37 (12%) | 7 (15%) | 0.47 |
|  |  | ≤10 | 262 | 37 |  | 282 | 39 |  |
| *mspA* | 11 | >11 | 33 (10%) | 0 (0%) | 0.008 | 13 (4%) | 1 (2%) | 1.0 |
|  |  | ≤11 | 282 | 55 |  | 308 | 45 |  |
| *pilC1* | 12 | >12 | 76 (27%) | 25 (48%) | 0.003 | 72 (23%) | 26 (57%) | <0.0001 |
|  |  | ≤12 | 210 | 27 |  | 239 | 20 |  |
| *pilC2* | 10 | >10 | 74 (25%) | 0 (0%) | <0.0001 | 102 (32%) | 17 (38%) | 0.499 |
|  |  | ≤10 | 218 | 43 |  | 214 | 28 |  |
| *opaA* | 8 | >8 | 9 (25%) | 3 (75%) | 0.073 | 16 (37%) | 23 (68%) | 0.011 |
|  |  | ≤8 | 27 | 1 |  | 27 | 11 |  |
| *opaB* | 8 | >8 | 16 (46%) | 2 (40%) | 1.0 | 24 (71%) | 20 (74%) | 1.0 |
|  |  | ≤8 | 19 | 3 |  | 10 | 7 |  |
| *opaD* | 13 | >13 | 8 (24%) | 4 (80%) | 0.025 | 22 (65%) | 24 (80%) | 0.265 |
|  |  | ≤13 | 26 | 1 |  | 12 | 6 |  |
| *opaJ* | 7 | >7 | 18 (49%) | 3 (50%) | 1.0 | 38 (97%) | 30 (100%) | 1.0 |
|  |  | ≤7 | 19 | 3 |  | 1 | 0 |  |

^1^ Numbers in brackets indicate the percentage of either invasive or carriage isolates with the higher repeat numbers; ^2^*p* values were derived from a two-tailed Fisher’s exact test.
